# Trace fossils associated with Burgess Shale non-biomineralized carapaces: bringing taphonomic and ecological controls into focus

**DOI:** 10.1098/rsos.172074

**Published:** 2019-01-16

**Authors:** M. Gabriela Mángano, Christopher David Hawkes, Jean-Bernard Caron

**Affiliations:** 1 Department of Geological Sciences, University of Saskatchewan, 114 Science Place, Saskatoon, Saskatchewan S7N 5E2, Canada; 2 Department of Civil, Geological and Environmental Engineering, University of Saskatchewan, 57 Campus Drive, Saskatoon, Saskatchewan S7N 5A9, Canada; 3 Department of Natural History-Palaeobiology, Royal Ontario Museum, 100 Queen's Park, Toronto, Ontario M5S 2C6, Canada; 4 Department of Ecology and Evolutionary Biology, University of Toronto, Toronto, Ontario M5S 3B2, Canada; 5 Department of Earth Sciences, University of Toronto, Toronto, Ontario M5S 3B1, Canada

**Keywords:** ichnology, exceptional fossil preservation, Cambrian, palaeobiology, palaeoecology, taphonomy

## Abstract

The association of trace fossils and non-biomineralized carapaces has been reported from Cambrian Lagerstätten worldwide, but the abundance, ichnodiversity, taphonomy and ecological significance of such associations have yet to be fully investigated. Two main end-member hypotheses are explored based on the study of a relatively wide variety of trace fossils preserved associated to *Tuzoia* carapaces from the middle Cambrian Burgess Shale in British Columbia. In the ecological *Tuzoia* garden hypothesis, the bacterially enriched surface of carapaces provides opportunities for intricate ecologic interactions among trophic levels. In the taphonomic shielding hypothesis, the trace fossil–carapace association results from preferential preservation of traces as controlled by compaction independent of any association in life. In an attempt to better understand the role of the carapace as a medium for preservation of trace fossils and to evaluate the effects of mechanical stress related to burial, a numerical model was developed. Results indicate that the carapace can shield underlying sediment from mechanical stress for a finite time, differentially protecting trace fossils during the initial phase of burial and compaction. However, this taphonomic model alone fails to fully explain relatively high-density assemblages displaying a diversity of structures spatially confined within the perimeter of carapaces or branching patterns recording re-visitation.

## 1. Introduction

The presence of trace fossils as a significant component of Burgess Shale-type deposits, better known for their exquisite preservation of soft-bodied animals, is rapidly gaining acceptance. Such trace fossils offer a dynamic picture of ecological interactions among organisms and with their environment, representing unquestionable evidence of *in situ* animal life [1–6]. There is still, however, considerable work to be done in outlining how trace fossils in Cambrian Burgess Shale-type deposits reveal taphonomic, palaeoecological and palaeoenvironmental information.

The association of trace fossils and non-biomineralized carapaces (e.g. [2,4,5]), while rare in post-Cambrian deposits, is particularly remarkable, and highlights synecological interactions and the emergence of new niches on the Cambrian sea floor. Moreover, the vast majority of trace fossils associated with carapaces in Burgess Shale-type deposits are small, typically less than 1.5 mm wide, representing the activity of small macrofaunal and meiofaunal elements [3,4]. Meiobenthic trace fossils have been documented from terminal Ediacaran ([7], but see [8]) and Cambrian [4] strata, but meiobenthic body fossils are generally rare [9,10] and mostly unknown in Burgess Shale-type deposits. The recent recovery of Small Carbonaceous Fossils (SCFs) from shallow marine siliciclastic deposits has revolutionized our understanding of the Cambrian meiobenthos, providing the oldest records of anostracan branchiopods, copepods and loriciferans, all of them with remarkably modern appearance [11,12]. In modern ecosystems, meiofaunal components, comprising both permanent and temporary meiofauna (i.e. juvenile macrofauna), play a key role at multiple levels, from engaging in complex interactions with bacteria (both encouraging their proliferation and using them as a food source) to direct energy transfer to higher levels via predation [13].

The association of trace fossils and non-biomineralized carapaces is a distinctive feature of several classic Cambrian Burgess Shale-type deposits, including the Burgess Shale itself (both classic and new localities (e.g. [3,14–16]), Sirius Passet (e.g. [4,17–20]), Kaili (e.g. [1,21]), Chengjiang [2], and the Barrandian area (e.g. [5,22])). On the other hand, trace fossils associated with non-biomineralized carapaces seem to be uncommon in the rest of the Phanerozoic (except for the Fezouata Formation of Morocco; P. Van Roy, personal communication, 2015), with very few records after the Cambrian [5].

In spite of all the above-noted investigations, detailed documentation of structures and further analysis of the potential implications of this peculiar trace–body fossil association has yet to be conducted. Whereas documented occurrences seem to have in common the association of trace fossils and non-biomineralized carapaces, taphonomic pathways and palaeoecological conditions may have differed significantly, recording a variety of interactions and processes. The aim of this paper is to provide a theoretical framework and an analytical model to evaluate the interplay of ecological and taphonomic factors in trace fossil–body fossil associations in Burgess Shale-type localities. We also attempt to further explore the significance of small macrofauna and meiofauna as components of the Burgess Shale community. Although the precise affinities of the producers are uncertain, feeding types can be reconstructed based on morphologic traits, and their synecological significance can be evaluated accordingly.

In this paper, we address whether the trace fossils associated with organic carapaces acted *only* as preservational agents (i.e. the ‘shield effect’) or if, at least in some cases, their presence was instrumental in promoting biogenic activity on the sea floor. We consider two end-member hypotheses, namely carapaces as attractors and promoters of biogenic activity (the ecological scenario or ‘*Tuzoia* garden’ hypothesis) versus carapaces as mediators across the fossilization barrier (the taphonomic scenario or ‘shield effect’ hypothesis). In order to analyse the role of stress and shear strain on trace-fossil distribution and morphology, and the production of carapace deformational features, a numerical model was developed.

A key issue to evaluate in these associations is the distinction between biologically and physically produced structures [2]. As we demonstrate in this study, whereas deformational wrinkles of the pliable carapace can mimic simple trace fossils, the fine morphology and complexity of diminutive structures unquestionably indicate biogenicity. The model presented here allows us to analyse numerically possible scenarios of compaction of the carapaces buried in horizontal and inclined positions, helping to visualize and explain deformational features affecting trace fossils and the carapace itself. In addition, we discuss how these occurrences may provide valuable evidence to reconstruct the colonization window, which in turn illuminates the role of environmental factors during deposition. This model is not intended to fully explain all trace fossils preserved in non-biomineralized carapaces. Rather, it aims to illuminate one potential taphonomic pathway. Further modelling may be necessary to address the diversity of morphologies of carapaces and potential pathways leading to trace fossils–non-biomineralized carapace associations.

## 2. Material and methods

*Tuzoia* has been chosen for the present analysis because it occurs in many Burgess Shale localities in the Canadian Rockies and other Cambrian Lagerstätten (e.g. [15,23–26]). Close to 50% (see electronic supplementary material, table S1) of the examined *Tuzoia* specimens at the Royal Ontario Museum (ROMIP) are associated with biogenic structures. We examined over 200 specimens of *Tuzoia* from the Burgess Shale collection deposited at the Royal Ontario Museum, Toronto, Canada (see electronic supplementary material, table S1). Material comes from two main stratigraphic intervals within the ‘thick’ Stephen Formation: (i) the Greater Phyllopod Bed from the Walcott Quarry Member, and (ii) the younger Raymond Quarry Member, in particular the so-called ‘*Tuzoia* layer’ or ‘*Tuzoia* bed’ [15,27,28], an approximately 3.5-metre-thick interval at the top of the Raymond Quarry Member. Some material also comes from the stratigraphically overlying ‘thin’ Stephen Formation at Stanley Glacier [3,16,29], including specimens recovered from claystone (*ca* 27–30 m above the base of the succession) and associated talus material. The preservation of only a couple of specimens of *Tuzoia* with soft parts suggests that freshly killed specimens were rarely entombed by mudflow deposits supporting a pelagic mode of life [15]. In the Burgess Shale it is common to find a gradient of fully articulated to fully disarticulated specimens of many nektobenthic soft-bodied species, thus implying different times of death and decay processes, but finding of mainly isolated carapaces is very rare [30]. Although it is possible that some specimens might represent carcasses, implying total decay of soft tissues prior to burial, most carapaces are assumed to represent exuviae in this study.

The stratigraphic architecture of the Stephen Formation is strongly controlled by the adjacent Cathedral Escarpment. As a result, the Stephen Formation is divided into ‘thin’ or ‘platformal’ Stephen Formation (up to 60 m thick), deposited shoreward of the escarpment in shallower water, and the ‘thick’ or ‘basinal’ Stephen Formation (around 300 m thick, = Burgess Shale Formation of [31]), which accumulated seaward of the escarpment [32]. Shoreline shifts during the middle Cambrian resulted in the formation of large-scale transgressive-regressive packages referred to as ‘Grand Cycles’ [33]. A typical cycle consists of outer detrital belt mudstone passing upwards into thin-bedded carbonate and culminating in massive carbonate [32,33]. Within this framework, the Stephen Formation represents the lower interval of the Stephen–Eldon Grand Cycle [32]. Smaller scale cycles (i.e. parasequences) composed of a lower mudstone and an upper carbonate-dominated unit can be detected only in proximal deposits of the thin Stephen Formation at Stanley Glacier [29], being essentially absent in more distal deposits recorded in Walcott Quarry and Raymond Quarry sections.

Additional observations were made on carapaces of the pelagic arthropods *Hurdia* and *Odaraia* and of the problematic benthic animal *Banffia* for comparative purposes*.* Many trace fossils are visible at first glance, but magnification reveals a higher number of biogenic structures that range into micrometric sizes. A Nikon SMZ 1500 stereomicroscope was used for detailed observations and a Nikon Digital Sight DS-Fi1 camera and Canon Eos 5D for microphotography. Drawings of selected bioturbated carapaces representing low- (less than 3% surface area covered), moderate- (3–10%) and high- (11–25%) density trace-fossil assemblages were created using Photoshop CS 6 (13.0). For each selected specimen, multiple digital photos were taken, illuminated from different directions at low or very low angles in order to highlight very low-relief structures. Photos were used as different background layers for the line drawings using Photoshop CS6 (13.0). Detailed observations under the microscope helped to improve the drawings. Percentages of area covered by trace fossils were calculated using ImageJ, following the methodology recently outlined by Cao *et al*. [34]. A suitable *Tuzoia* carapace (part and counterpart), showing clear evidence of biogenic structures, was selected to perform elemental maps (see electronic supplementary material, figure S2). Elemental maps are used to detect any variation in mineralogy between biogenic structures and the matrix.

**Figure 1.**
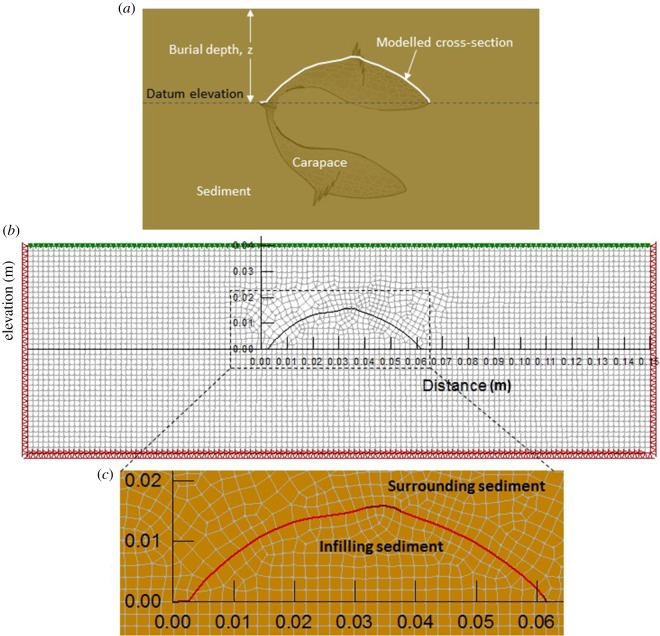
Cross-sectional view of *Tuzoia* carapace and model geometry. (*a*) Illustration showing complete carapace after burial in sediment. The hinge (dorsal margin) is on the left and the ventral margin on the right. Carapace shape based on fig. 1.4 in Vannier *et al*. [15]. (*b*) Model cross section generated using SIGMA/W (GEO-SLOPE International Ltd., 2012), showing upper half of carapace at its maximum height (i.e. about halfway along its length). Horizontally-orientated upper half (base case—shown here) and inclined (‘tilted’) scenarios discussed in the text. The dimensions shown are in metres; as such, the width of the carapace shown is nearly 0.06 m (60 mm). The pairs of red triangles along the lower boundary represent a zero-displacement boundary condition. The red triangles along the lateral boundaries denote a boundary condition in which displacements are allowed in the vertical direction, but not in the horizontal direction. This is in line with previous interpretations that compaction of the body and surrounding matrix proceeded in the same way (i.e. without lateral expansion of the fossils; [35]). At its centre, the carapace is overlaid by approximately 0.025 m (25 mm) of sediment. The green arrows along the upper boundary denote additional load applied to the upper surface. This additional load is added incrementally during modelling, to emulate progressively deeper burial of the carapace. (*c*) Magnified view of the modelled carapace. The quadrilateral and triangular mesh elements show the spatial discretisation of the sediments within and surrounding the carapace. The red line that traces the carapace denotes the structural (beam) elements used to emulate the mechanical behaviour of the carapace. The darker shades of red at the ridge and near the margins denote zones where carapace thickness was assumed to be slightly increased (see electronic supplementary material, Numerical Model for details).

To understand better the role of stress and shear strain in carapace deformation and potential preservability of trace fossils, a numerical model was constructed using SIGMA/W (GEO-SLOPE International Ltd., 2012), a commercial finite-element software product developed to perform stress and deformation analyses of earth structures. The model was used to assess deformation and stresses for a convex side-up *Tuzoia* valve. The basic premise of the model geometry is illustrated in figure 1*a*, which shows both valves of a carapace which has been deposited laterally. Although the exact orientation of the carapace would vary from one specimen to the next, the base case scenario illustrated here is one in which the upper valve is orientated horizontally. The datum elevation for modelling was chosen as the horizontal surface upon which the dorsal and ventral margins of the ‘arch’ defined by the upper valve make contact with the underlying sediment (other scenarios in which the modelled cross-section has different orientations are discussed later). Compaction will ultimately juxtapose the valves on top of each other. Based on the abundance of associated shell material, some carapaces may have remained on the sediment surface for some time. During this time the carapace may have been empty or only partially infilled by sediment and, in principle, colonization could have taken place. However, the preferred scenario (i.e. short transport and entombment by mass flows, see [15] and Discussion), would result in the destruction of any previously formed biogenic structures and infill of the space between the valves with sediment. For simulation purposes, the upper valve was included in the model because of its convex side-up orientation, which would have allowed it to serve as an arch and redistribute stresses beneath and surrounding the valve in such a way as to affect the preservation of traces. The lower valve, being orientated concave side-up, likely had less effect on stress redistribution and it was not included in the model. Although only *Tuzoia* carapaces were modelled, the basic assumption is that any strongly convex side-up skeletal part affected by compression will follow a similar compaction pathway.

The model considers a two-dimensional, cross-sectional view of the carapace, with mechanical properties drawn from data published for chitin-based materials (electronic supplementary material, table S1). The carapace shape was taken from figs 1–4 of [15], and the valve height of 60 mm was chosen as a representative value based on fig. 26 of [15]. Figure 1*b* shows the model domain used for the base case scenario, with figure 1*c* showing a magnified view of the modelled upper valve of the carapace. The boundary conditions shown in figure 1*b* follow standard geotechnical modelling practice, and the boundaries were placed at sufficient distance from the carapace to mitigate their influence on stresses and deformations local to the carapace.

Assumptions made for the modelling include the following (see electronic supplementary material): (i) 2-dimensional, plane strain geometry, (ii) drained loading (i.e. no generation of excess pore pressure during burial), (iii) linear elastic-plastic constitutive behaviour for sediment, (iv) linear elastic behaviour for the structural elements used to represent the *Tuzoia* carapace, (v) constant carapace thickness, and (vi) mechanical properties of the carapace remaining constant over time (i.e. degradation processes acting on carapaces are neglected).

In addition, the weight of overlying sediments is considered to be the main source of mechanical deformation of the carapace and trace fossils. This process is assumed to have occurred contemporaneously with sedimentation or immediately after (i.e. synsedimentary). The model is intended to demonstrate the patterns of stresses and deformations in the early stages of burial and compaction, up to the point where the carapace begins to fail (e.g. by buckling under compressive stress, resulting in the development of wrinkles). Late brittle deformation related to tectonic processes is evident in some specimens, but those structures are unrelated to the present study. In the model we assume negligible effect of any relict soft tissue on mechanical stiffness or strength of the carapace since most specimens probably represent moults.

## 3. Morphological variability of trace fossils associated with *Tuzoia* carapaces

We use the conceptual framework of ichnology [36–40] to analyse trace fossils associated with non-biomineralized carapaces. Most studies involving trace fossils in Burgess Shale-type deposits do not necessarily use standard terminology, making limited use of the available descriptive and conceptual tools (see [5] for an exception). In this paper, we attempt an ichnotaxonomic approach to facilitate comparisons among body fossil–trace fossil associations across Burgess Shale-type deposits, but also between Burgess Shale-type and other Phanerozoic ichnofaunas. Ichnotaxobases are used in trace-fossil characterization and, whenever possible, an ichnotaxonomic assessment of biogenic structures is attempted (based on well-preserved specimens).

**Figure 2.**
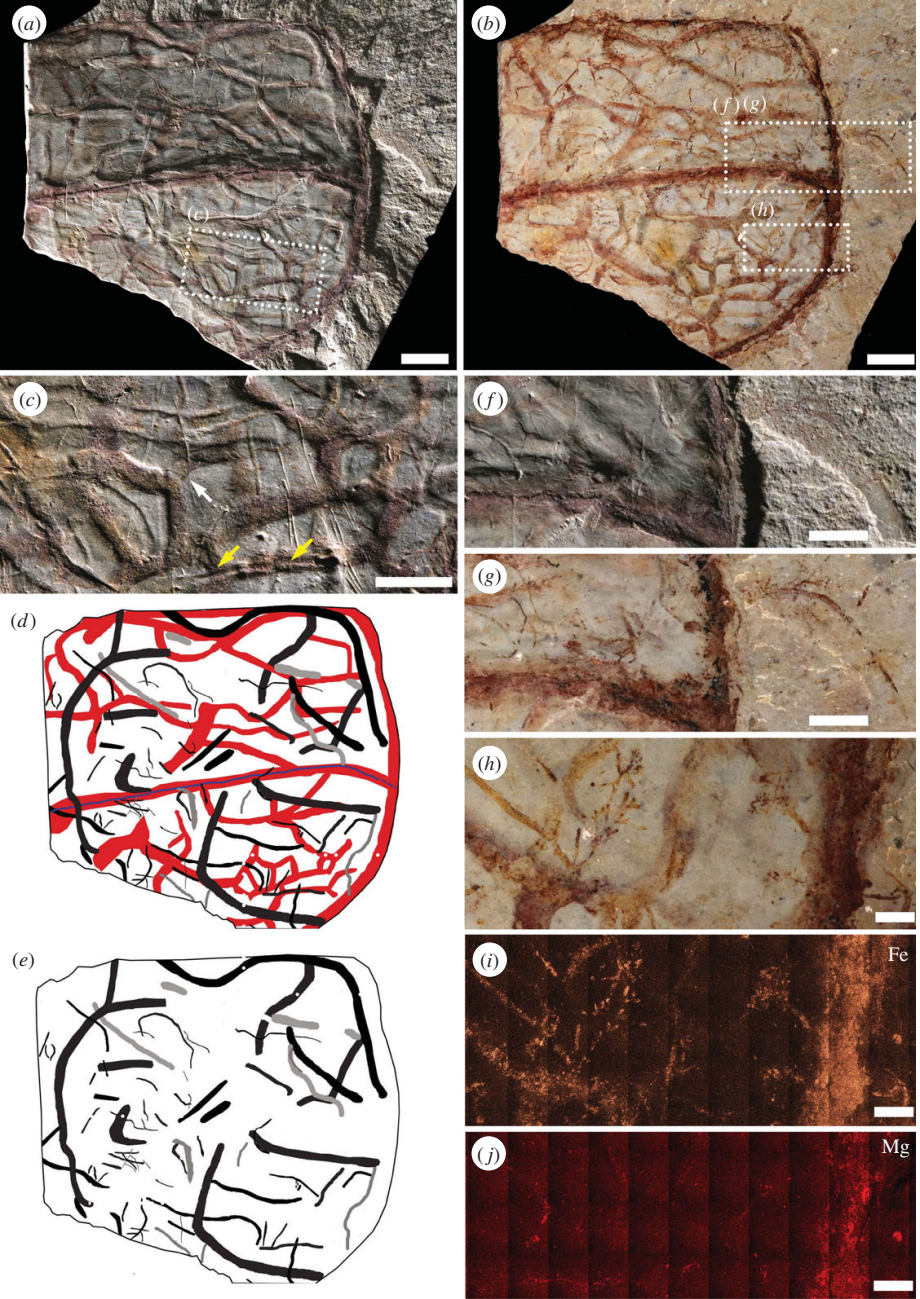
(*Overleaf*.) Densely bioturbated and poorly-preserved *Tuzoia* carapace showing no evidence of original polygonal structure, ROMIP 57367, Stanley Glacier. (*a*,*b*) Specimen photographed using low angle (*a*) and cross-polarized (*b*) lighting conditions. The apparent intricate network of interconnected burrows, suggesting a complex gallery network, is partially a taphonomic artefact. Some skeletal elements (typically the margins and median ridge), cuticle wrinkles, trace fossils, and other branching biogenic structures are stained in iron oxides, while many burrows are compressed on top of each other. (*c*) Detailed view of false branching (white arrow) in heavily iron-stained burrow. Notice the iron-stained burrows mimicking a polygonal network and the small, straight, high relief trail (*Helmintoidichnites tenuis*) running parallel to a stained burrow (yellow arrows). (*d*) Interpretative drawing showing all elements stained by iron oxides (red), non-stained trace fossils (black) and compression artefacts (grey). Notice that the iron oxides are not confined to trace fossils and many trace fossils are not iron-stained. (*e*) Interpretative drawing showing only those elements that are not iron-stained. (*f*) Close-up showing trace fossils affected by compaction (i.e. very flat and irregular boundaries) that are not preferentially highlighted by iron oxides*.* (*g*,*h*) Close-ups showing branching biogenic structures of uncertain origin (?*Pilichnus* isp.). These structures seem to grow on top of burrows and display very distinct boundaries suggestive of a biogenic origin (*contra* simple mineral growth). (*i*,*j*) Elemental maps showing that Fe and Mg occupy both margins of the carapace and some biogenic structures suggesting a taphonomic artefact. All scale bars are 1 cm, except 0.2 cm in (*h–j*) and 0.5 cm in (*c*,*f*,*g*).

The studied trace fossils are predominantly horizontal, displaying minimal penetration into the shale (typically less than 2.5 mm). Some structures, however, do cross submillimetric laminae, moving from one horizontal level to another. Trails and burrows ranging in diameter from micrometric to about 1.5 mm are common features associated with carapaces of *Tuzoia* and other non-biomineralized carapaces (e.g. *Hurdia*) in Burgess Shale localities. Structures wider than 3 mm are extremely rare. Most trace fossils fall into the architectural category of simple horizontal trails *sensu* [40], being followed in abundance by horizontal branching burrows and actively filled pelleted horizontal burrows. Aligned disconnected mounds have been observed in several specimens, but the irregular distance between mounds and absence of proof of internal connection raises doubt about their precise affinity (but see an alternative interpretation by [5], fig. 6 ‘*Treptichnus*-like’). Although a dense and complex burrow network seems to be associated with some poorly-preserved specimens of *Tuzoia*, locally mimicking preservational variants of *Arachnostega*, detailed examination indicates that the apparent galleries are the product of compaction and weathering (i.e. a taphonomic artefact). In many cases, compressed, partially collapsed structures highlighted by iron oxides result in colourful, intricate pseudo-networks (figure 2*a–i*).

### 3.1. Straight-to-curved simple burrows and trails

**Figure 3.**
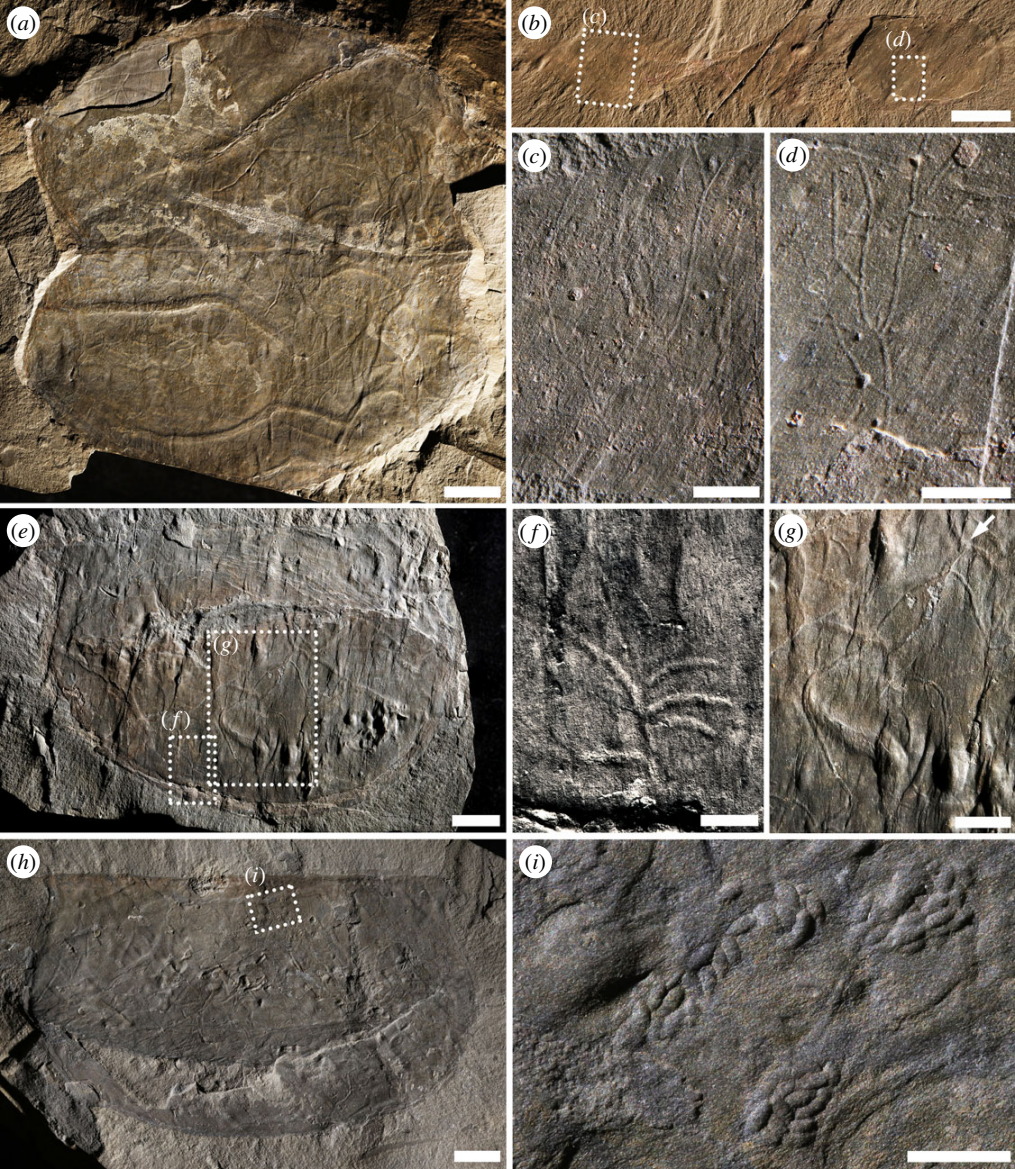
Diversity of trace fossils associated with non-biomineralized carapaces (*Tuzoia*) and soft-bodied animals (*Banffia*) from the Burgess Shale, Fossil Ridge. (*a*) Simple straight-to-curved trails assigned to *Helminthoidichnites tenuis*. Notice the different sizes and cross-sectional morphologies. ROMIP 57444, *Tuzoia* Bed. (*b–d*) Tree-like, delicate branching structures assigned to *Pilichnus* isp. preserved on both the posterior (*c*), and anterior sections (*d*) of the problematic animal *Banffia constricta*. ROMIP 49893, Collins Quarry. (*e–g*) ROMIP 57409, *Tuzoia* Bed; (*e*) Overall view; (*f*) Detail showing multi-branched burrow displaying curved segments from axial structure, cf. *Pilichnus* isp.; (*g*) Detail showing a long specimen of *Helminthoidichnites tenuis* displaying 90° turns cross-cutting strongly compressed, interconnected burrows branching at 120° (white arrow). Notice the contrasting relief of different structures, most likely reflecting constructional differences. (*h*,*i*) ROMIP 57524, *Tuzoia* Bed, (*h*) Overall view; (*i*) Pellet-infilled burrows, *Alcyonidiopsis* isp. Notice closely packed ovoid pellets packed in finger-like blind short burrows. All scale bars are 1 cm, except 0.2 cm in (*c*,*d*,*f*,*i*) and 0.5 cm in (*g*).

**Figure 4.**
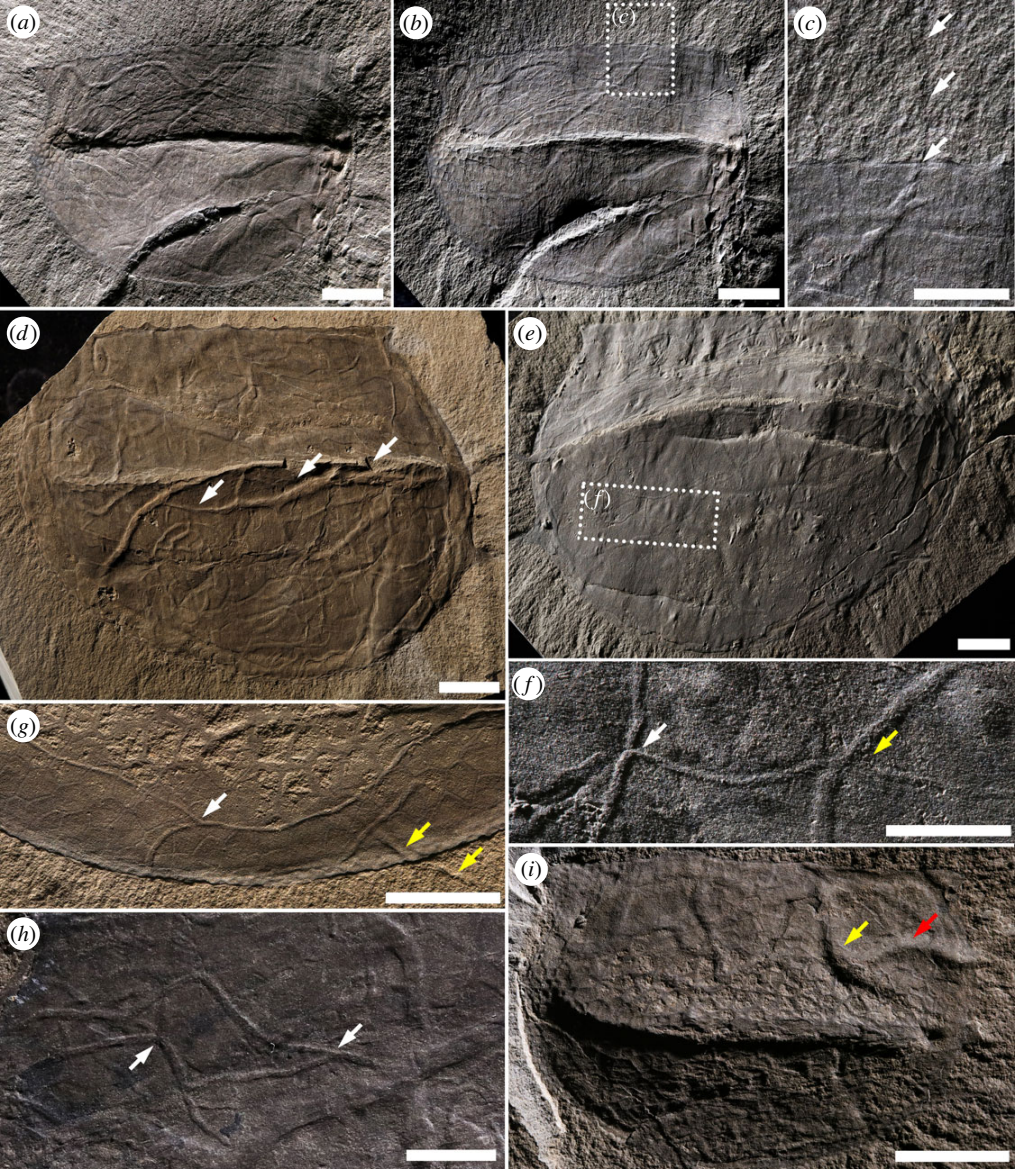
Trace-fossil–carapace interactions. All *Tuzoia* carapaces from the *Tuzoia* Bed, Fossil Ridge. (*a–c*) *Helminthopsis hieroglyphica* following closely the dorsal hinge line of *Tuzoia*, ROMIP 57503; (*a*,*b*) Overall view, (*b*) using opposite low angle lighting compared to (*a*), (*c*) close-up showing overcrossing of trace fossils within the carapace and possible extension of one structure outside the carapace (white arrows). (*d*) High density of trace fossils showing common overcrossing and trace-fossil morphologic distortion. Notice drastic change in width along structure due to compression (white arrows), ROMIP 57419. (*e*,*f*) ROMIP 57454; (*e*) Overall view, (*f*) close-up showing secondary successive branching (white arrow) and overcrossing (yellow arrow). (*g*) Branching burrows following the ventral margin of *Tuzoia*. Notice one simple burrow seems to cross the outline of the carapace into the surrounding matrix (yellow arrows). See figure 6*b* for overall view of specimen, ROMIP 57511. (*h*) Secondary successive branching (white arrows). Notice the sharp 90° turns along the pathway of structures. See figure 6*c* for overall view of specimen, ROMIP 57496. (*i*) Small specimen of *Tuzoia* showing large trails overcrossing (yellow arrow) and one of them exiting the carapace (red arrow); ROMIP 57489. All scale bars 1 cm, except 0.5 cm in (*f*,*h*).

**Figure 5.**
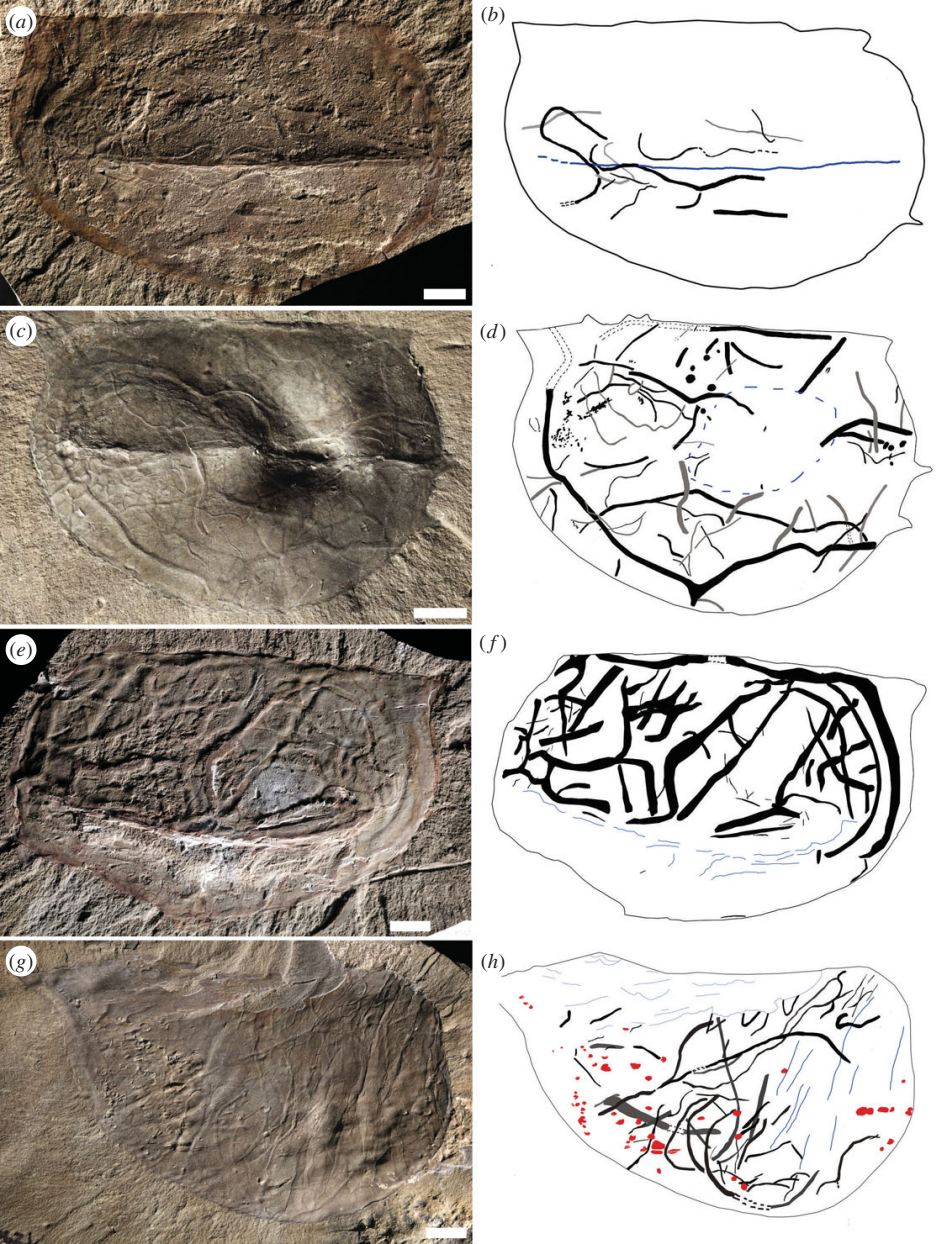
Trace-fossil distribution maps in *Tuzoia:* ecological versus taphonomic control. (*a*,*b*) Low-density assemblage (ImageJ = 3%). Notice trace fossils are mostly concentrated around the middle ridge area. ROMIP 57366, Stanley Glacier. (*c*,*d*) High-density trace-fossil assemblage (ImageJ = 13%) showing widespread distribution (i.e. non-preferential). Notice long structure following anterior and ventral margin. ROMIP 57394, *Tuzoia* Bed, Fossil Ridge. (*e*,*f*) High-density trace-fossil assemblage (imageJ = 23%) showing no bioturbation below the middle ridge and the ventral margin. The presence of wrinkles in this area suggests that absence of trace fossils is likely a taphonomic artefact (see text). ROMIP 57370, Stanley Glacier. (*g*,*h*) Moderate-density trace-fossil assemblage (ImageJ = 10%). Strongly-deformed specimen showing trace fossils and abundant shell material trapped below the carapace (red). Notice distribution of trace fossils and wrinkles. Deformational features (i.e. wrinkles) are present in the posterior area (right) and close to the dorsal hinge (anterior) where trace fossils are essentially absent, most likely due to a taphonomic overprint (see text). ROMIP 57406, *Tuzoia* Bed, Fossil Ridge. All scale bars are 1 cm.

These structures are the most common trace fossils associated with carapaces. Most of the trails are straight to curved (figures 2*e*, 3*a*,*e*,*g*, 4*a–d* and 5*a*,*b*); semicircular paths (i.e. arcs) are also relatively common (figure 5*g*,*h*). Width is typically micrometric to millimetric (0.1–2.8 mm). Interestingly, larger trails tend to exhibit flatter morphology (figures 2*c* and 3*a*) and a sub-square cross-sectional view. Sharpness and width can change quite drastically along the length of the trail (figures 2*b* and 4*d*), implying some subtle vertical component in the movement or as a result of moving into areas of different stress/shear strain below the carapace. In relatively dense trace-fossil assemblages, overcrossing is common (figures 2*e* and 4*c*,*d*,*h*). Length varies from a few millimetres in incompletely preserved structures to 14 cm. Some longer trails follow the margin of the carapace (figures 2*a*,*d*, 4*a* and 5*e*,*f*) or the median ridge (figure 4*d*). Straight-to-curved simple trails are here included within *Helminthoidichnites tenuis*. Here, a trail is considered a continuous structure recording locomotion, although other activities, such as feeding, may have often taken place in them as well [38]. Accordingly, many trails are not simple locomotion structures (repichnia), but rather record combined locomotion and feeding (i.e. grazing), and so are included within the ethological category of pascichnia. In marine settings these structures are commonly produced by a wide variety of vermiform metazoans, although *Helmintoidichnites*-like trails have been reported as produced by giant protozoans [41]. *Helminthoidichnites* is included within the architectural category of simple horizontal trails [40]. Chlupáč & Kordule [23] documented trails of vermiform animals in several arthropod carapaces, including *Tuzoia*, and tentatively placed them in *Helminthopsis* and *Gordia*. These trace fossils are here included within *Helminthoidichnites*, but some of them seem to represent wrinkles (compare [23], fig. 4 with [5], fig. 5*E*). Wang *et al*. [1] illustrated *Gordia marina* associated with bivalved arthropods in the Kaili biota. However, lack of self-overcrossing and general morphology of these structures is suggestive of either *Helminthoidichnites* or *Helminthopsis* (except for fig. 4*F* and *I*). *Helmintoidichnites*-like structures have also been documented in association with non-biomineralized carapaces from Chengjiang [2].

### 3.2. Meandering and sinusoidal trails

These structures are less common than straight or curved trails, but are commonly associated with them. Within this group we find irregular and regular sinusoidal structures 0.2–2.5 mm wide and up to 45.7 mm long. Irregularly meandering trails are here included within *Helminthopsis tenuis* or *Helminthopsis hieroglyphica* (figure 4*a*), and regular sinusoidal structures fit best within *Cochlichnus anguineus*. Both ichnogenera are considered grazing trails (Pascichnia). Marine examples of *Helminthopsis* are typically regarded as produced by vermiform animals [42]. In marine settings, *Cochlichnus* is commonly considered as produced by free living nematodes, which represent the dominant meiofaunal group in muds, and are strongly linked to the detritus/bacteria food chain [13]. *Helminthopsis* and *Cochlichnus* are also included within the architectural category of simple horizontal trails [40]. Mángano ([3], fig. 2*C*) documented *Cochlichnus* associated with *Hurdia* at Stanley Glacier. Mikuláš *et al*. [5] included more or less regularly meandering trails within ‘*Cochlichnus*-like structures’. Some of the illustrated specimens seem to conform to this ichnotaxon ([5], fig. 4*B*), but others are too fragmentary or do not show the regular sinusoidal nature of *Cochlichnus* ([5], fig. 4*A*,*E*).

### 3.3. Pellet-infilled structures

These structures are characterized by the presence of a massive infill of ellipsoidal tiny pellets (0.4–0.7 mm long; 0.1–0.3 mm wide). No constructional wall or lining is observed, and the boundary of the structure is locally defined only by aligned packed pellets (figure 3*i*). Although in most cases there is no clear organization of the pelletoidal infill, groups of two side-by-side pellets are locally present. Burrow width is 0.5–1.8 mm. Maximum length is about 8 mm. Most burrows seem to reflect some limited up and down movement through laminae (i.e. not being completely confined to one lamina), showing limited horizontal length and locally circular cross sections. Clusters of pellets can also be dispersed (i.e. not confined within a distinct burrow), but distributed over a restricted area. Massive pellet accumulations and tubular pellet-infilled structures have been commonly assigned to *Tomaculum* in the literature (e.g. [42,43]). However, several authors have revised the taxonomy of pellet-infill burrows concluding that *Tomaculum* is a junior synonym of *Alcyonidiopsis* [44–46]. Accordingly, the pellet-infilled structures analysed here are included within *Alcyonidiopsis longobardiae.* Exquisitely preserved three-dimensional pellets are composed of sediment rather than organic material, suggesting deposit feeders as opposed to predators and scavengers. The fact that pellet morphology is not round, but typically ellipsoidal (figure 3*i*), is suggestive of an arthropod or polychaete producer [47]. Recent polychaete pellets display striking similarity to the analysed material (cf. [48]). *Alcyonidiopsis* is included in the architectural category of simple, actively filled (pelletoidal) horizontal burrows [40]. Pellets and pelletized structures associated with non-biomineralized carapaces were reported from Chengjiang by Zhang *et al*. ([2], fig. 3*C*,*D*), although no description of these trace fossils or faecal pellets were provided. Larger pellet-infilled structures not belonging to *Alcyonidiopsis* have been described from other Cambrian Lagerstätten [18,21]. Faecal pellets forming part of the infill of large burrows in association with an eldoniid were reported and illustrated from the Kaili Biota (fig. 8 in [21]). A ‘spicular’ or nodose texture associated with relatively large burrows (*ca* 2.5 mm) in Sirius Passet was suggested as possible pellets of actively infilled burrows [18].

### 3.4. Delicate, filament-like branching structures

These structures exhibit a very distinctive morphology. Filament-like structures display a diagnostic, typically dichotomous branching morphology suggestive of primary successive branching (figure 3*c*,*d*). Some more complex, multiple-branching morphologies are occasionally observed (figure 3*f*). These tree-like structures are formed by several short, curved segments that depart from the axial probe of similar width. Burrow width is 0.8–1.2 mm. Maximum length of axial probe is 13.5 mm. Filament-like structures are typically attributed to the feeding trace fossil *Pilichnus.* In modern shallow-marine environments, structures similar to *Pilichnus* are formed by polychaetes [49]. *Pilichnus* corresponds to the architectural category of horizontal branching burrow systems [40]. Crenulated, filament-like branching structures associated with non-biomineralized carapaces at Sirius Passet have been tentatively assigned to the ichnogenus *Pilichnus* [4]. However, fine morphological details, such as the distinctive crenulated path and long axial probes associated with terminal or lateral tree-like elements, seem to be absent in the analysed material and indicate some differences between delicate branching structures at Sirius Passet and Burgess Shale. Further ichnospecific treatment of these structures awaits the recovery of additional material. Mikuláš *et al*. ([5], fig. 5*C*) described very thin, dichotomous branching structures (40°–90°) as ‘*Pilichnus*-like’.

### 3.5. Interconnected burrows

These trace fossils are composed of straight to curved segments, 1.3–2.6 mm in diameter, which bifurcate (i.e. simultaneous branching) or interconnect at variable angles (i.e. secondary successive branching) with typical angles of 60°–90° (figure 4*f–h*). The main challenge for recognizing these structures is to distinguish branching (either simultaneous or secondary successive) from overcrossing (i.e. false bifurcation *sensu* [37]). In crowded specimens, this distinction may not always be possible. However, in less dense trace-fossil assemblages, detailed analysis of burrow intersections may reveal the type of branching (figure 4*f–h*) and clearly distinguish it from overcrossing (figure 4*f*,*i*). The presence of kinks at the intersection of many of these burrows seems to be a distinctive feature. Flat morphologies, particularly evident in larger structures, suggest these burrows were most likely kept open and passively infilled, suggesting reuse by the trace makers. Only very rarely have expansions been observed at branching points (figure 5*c*,*d*); these are reminiscent of ‘turnarounds’ observed in fossil and modern crustacean burrows [50]. Structures assigned to this group are not so straightforward in terms of identification and ethological interpretation. Morphological features of these ‘interconnected burrows’ display affinities with the ichnogenus *Multina* [42,51]. Based on functional morphology and analogy with modern structures, these trace fossils are ethologically interpreted as recording repeated grazing on bacteria growth on mucus-rich trails or linings and are attributed to vermiform organisms. *Multina* is included in the architectural category of horizontal branching burrow systems [40]. Similar so-called ‘bifurcating or branching burrows’ were described from Chengjiang by Zhang *et al*. ([2], fig. 2*A*,*D*,*F*,*G*), and Peel [18] and Mángano *et al*. [4] from Sirius Passet.

### 3.6. Annulated burrows

These structures consist of horizontal, curved burrows characterized by distinct transverse annulations (electronic supplementary material, figure S1*a*,*b*). No wall is apparent. Burrow fill seems to be identical to the host rock. Burrow width is 0.8–1.2 mm. Maximum length is about 350 mm. Annulations are 0.2–0.4 mm wide. Backfilled, annulated burrows have been assigned to a number of ichnotaxa, including *Planolites annularis* and the various ichnospecies of *Taenidium*, among others [52–54]. Limited material prevents making a definitive ichnotaxonomic assignment. The transverse annulations in all probability record an active backfill, and these trace fossils are interpreted as representing feeding activities by vermiform animals. Interestingly, Kulkarni & Panchang [48] recorded modern annulated burrows produced by polychaetes and interconnected to *Alcyonidiopsis*-like structures, indicating a common producer for both structures. Annulated structures most likely belong to the architectural category of simple actively filled (meniscate) horizontal to oblique structures [40]. Annulated burrows associated with non-biomineralized carapaces seem to be relatively rare in Burgess Shale-type deposits, but were recorded in Sirius Passet [4].

### 3.7. U-shaped burrows

These structures consist of highly convex segments representing the base of U-shaped vertical burrows (electronic supplementary material, figure S1*c*). Burrow fill is identical to the host rock. Burrow width is 1.3–2.6 mm. Length is 6.1–8.4 mm. Although superficially resembling simple burrows, the high convexity points to a U-shape structure. They have been identified in a single specimen recovered from the Walcott Talus, being distributed all over the sample both in association with *Tuzoia* and in the surrounding sediment. Similar U-shaped burrows have been assigned to the ichnogenera *Arenicolites* or *Diplocraterion*. Differentiation of these two ichnotaxa is based on the presence of spreite in the latter [55]. The lack of cross-section views prevents a definite ichnotaxonomic assignment. These burrows most likely represent dwelling burrows. Potential producers are vermiform organisms (e.g. polychaetes, priapulids) or crustaceans [37,55]. These structures correspond to the architectural category of vertical single U- and Y-shaped burrows [40]. Stratigraphic surfaces displaying larger, high density assemblages of U-shaped burrows have been recorded in Raymond Quarry [56]. No carapaces or soft-body fossils, however, have been documented in these bioturbated levels of the Raymond Quarry [56].

## 4. Quality of preservation, distribution and density of trace fossils associated with *Tuzoia* carapaces

**Figure 6.**
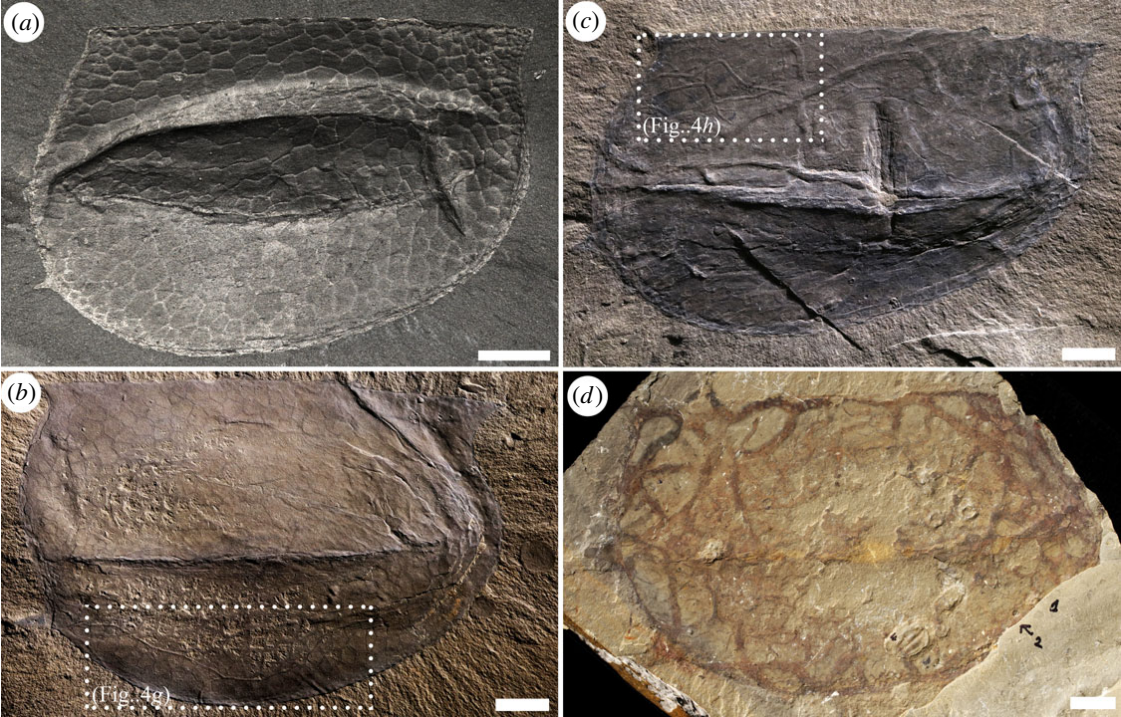
Preservational range of *Tuzoia* carapaces and degree of fidelity of biogenic structures. (*a*) Well-preserved *Tuzoia* carapace showing exquisite detail of the organic polygonal structure and absence of trace fossils. This type of specimen commonly displays a characteristic glossy surface and, in some cases, a dark carbon film. ROMIP 57313, Raymond Quarry, Fossil Ridge. (*b*,*c*) Moderately preserved *Tuzoia* carapaces*;* the organic polygonal structures are locally observed, but trace fossils are clearly visible displaying a variety of morphologies and sizes. Although structural elements of the carapace may be locally quite well-preserved, bioturbated areas and common shell concentrations result in more irregular surfaces; (*b*) Weakly bioturbated carapace, ROMIP 57511, *Tuzoia* Bed, Fossil Ridge. Close-up area, see figure 4*g*; (*c*) Moderately bioturbated carapace, ROMIP 57496, *Tuzoia* Bed, Fossil Ridge*.* Close-up area, see figure 4*h*; (*d*) At the other extreme of the preservational spectrum, taphonomic artefacts prevent ichnotaxonomic assessment in most cases, ROMIP 57369, see also figure 2. All scale bars are 1 cm.

*Tuzoia* specimens from the Burgess Shale reveal a wide range of preservational variants both within and between assemblages. Because it is difficult to provide a quantitative assessment of preservation, we provide a qualitative estimation that, although simplistic, permits a focus on observable elements: degree of preservation of the polygonal structure of the carapace, distribution of compression artefacts, and presence/absence of iron oxides. There is a wide range in the degree of compaction. Some specimens exhibit obvious volume and are symmetrically or asymmetrically compressed. Others are completely flat, display a relatively smooth surface and lack of discrete deformational features. There does not seem to be a correlation between three-dimensionality and the presence or absence of trace fossils. Based on the analysed material, three main *Tuzoia* preservational variants can be characterized. These variants define a gradient of preservations. At one end of the spectrum, carapaces show no or limited compression artefacts (i.e. wrinkles, if present, are localized), and the polygonal structures are pristinely preserved in many cases with remnants of carbonaceous film. In the Walcott Quarry, such specimens show a characteristic reflective, dark grey smooth surface, and appear, for the most part, devoid of trace fossils (figure 6*a*). In the stratigraphically younger Raymond Quarry, moderately to well-preserved specimens display partial preservation of the original polygonal structure and little (less than 3%) to moderate (3–10%) bioturbation. In this intermediate state of preservation, many *Tuzoia* specimens still exhibit a smooth surface limited by the edges of the carapace. ‘Moderately preserved’ *Tuzoia* reveal well-preserved, distinct trace fossils (figure 6*b*,*c*). These specimens are typically not severely compromised by deformation, and late diagnetic processes and/or weathering, if present, are not pervasive. At the other end of the spectrum, some heavily wrinkled and/or densely bioturbated specimens present a rough, typically irregular surface, and the polygonal structure is typically absent (figures 2*a*,*b* and 6*d*). *Tuzoia* carapaces in this state of preservation from Stanley Glacier are also rather flat, whereas those from the *Tuzoia* Bed assemblage tend to be more three-dimensional and show strong symmetrical or asymmetrical deformation. Typically, specimens in this latter category show different degrees of weathering; iron staining tends to outline skeletal morphological features (e.g. carapace margin, spines, processes and lateral ridges), but also many biogenic structures and deformational mechanical features (e.g. wrinkles and ruptures), suggesting a non-selective late diagenetic process [57]. Assessment of the density of trace fossils and evaluating morphological features with ichnotaxonomic significance (e.g. type of bifurcation) can be challenging and not always possible, as deformation results in superposition of structures and common artefacts.

Although the whole range of preservational variants was studied, only relatively well to moderately preserved *Tuzoia* specimens were considered to evaluate ichnotaxobases for ichnotaxonomic determination and reliable descriptive characterization. Poorly preserved *Tuzoia* can be misleading during the evaluation of trace-fossil density and, particularly, trace-fossil diversity. These specimens, however, are still highly informative and play a significant role in understanding the taphonomic overprint and the colonization window.

In most sedimentary deposits, trace fossils are preserved as semi-reliefs, either on the top or base of beds (e.g. cast at lithological interfaces) or they are preserved as full reliefs within beds. Trace fossils in the analysed deposits, however, do not necessarily follow the classic rules of ichnologic taphonomy. Most laminae are undisturbed and lithological interfaces are quite sharp and unbioturbated. Contrasting with mostly ‘unbioturbated’ cross-sectional views, however, bedding surfaces hosting non-biomineralized fossils can reveal a bioturbated seascape, characterized by the heterogenous distribution of trace fossils. This patchy distribution of trace fossils highlights the presence of *Tuzoia* or other cuticles on stratigraphic surfaces.

Detailed observations of the distribution of these biogenic structures show that many trace fossils abruptly interrupt their course coincident with the margin of the carapace and only rarely can be tracked crossing into the surrounding sediment (figure 4*c*,*g*). Such trails and burrows have diffuse boundaries, are discontinuous, being only locally visible, and display extremely low relief and an infill indistinguishable from the host rock (see electronic supplementary material, figure S1*c* for an exception). Although trace fossils can be present in very low densities (less than 3%) in the surrounding sediment, clearly the largest density and diversity of trace fossils are confined within the perimeter of the carapace. The trace maker's apparent preference to live around or use resources associated with the carapace is suggested by the careful attention of most biogenic structures to the carapace surface, including long structures that closely follow the dorsal hinge or ventral margin of the valve and occasional local inflections on the carapace margin (figures 2*a*,*d*, 4*a* and 5*e*,*f*). Unfortunately, due to the size of rock samples—museums mostly cut around fossil specimens—it is rarely possible to provide a precise BP-BI (bedding plane bioturbation index [58]) for the host sediment surface. However, based on observations in the field and in a few larger samples, BP-BI is 1 in almost all measured cases, and only exceptionally 2 (see, for example, text-figure 3 in [3]).

In terms of the relation between bioturbated versus unbioturbated *Tuzoia*, there are significant differences among the three stratigraphic intervals analysed (Greater Phyllopod Bed at Walcott Quarry, Raymond Quarry *Tuzoia* Bed, and Stanley Glacier). *Tuzoia* specimens from the Greater Phyllopod Bed in the Walcott Quarry (*N* = 27) do not contain associated trace-fossil assemblages. In fact, only one *Tuzoia* exhibits isolated *Helminthoidichnites*, representing only 3.7% of the total number of *Tuzoia* specimens (see electronic supplementary material, table S1). In addition, a few other non-biomineralized skeletal elements (e.g. *Odaraia*) may display localized *Alcyonidiopsis*. Particularly remarkable is the presence of delicate *Pilichnus*-like structures in *Banffia constricta* (cf. [59] and figure 3*f*). In contrast, over half of the *Tuzoia* carapaces from the Raymond Quarry *Tuzoia* Bed (*N* = 168) contain biogenic structures (52.4%). However, over 70% of these *Tuzoia* population have less than 3% of their total surface bioturbated, and only about 10% of bioturbated specimens record high densities (see electronic supplementary material, table S1). At Stanley Glacier over 75% of the analysed *Tuzoia* (*N* = 21) display biogenic sedimentary structures with the bioturbated percentage area typically between 8% and 25% (moderate to high densities).

Distribution maps of trace fossils and deformational features (i.e. wrinkles, sediment extrusion) reveal some patterns. Trace fossils on carapaces displaying low density of trace fossils less than 3% (typically recorded by a few simple trails) display no preferred location on the carapace (i.e. trace fossils can be localized in anterior, posterior, lateral ridge, hinge or ventral areas) and occasionally these structures can be traced in and out of the carapace (e.g. figure 4*i*). In slightly higher densities, typically 3–5%, trace fossils may display some preferential distribution, such as around the ridge area (figure 5*a*,*b*) or following the dorsal or ventral margin of the carapace at some distance (figure 4*a*,*b*,*g*). If not strongly deformed, distribution patterns in high-density trace-fossil assemblages (11–25% covered area) seem to occupy evenly the whole area of the carapace (figures 4*d* and 5*c–h*), being present in the anterior, posterior, hinge and ventral margin areas, in many cases long trails and burrows following the margin of the carapace. However, *Tuzoia* specimens displaying localized severe deformation typically show non-bioturbated areas (e.g. middle ridge to ventral margin or middle ridge to hinge line) adjacent to areas exhibiting moderate to high density of structures (figure 5*e–h*). This irregular distribution pattern can be explained as a taphonomic overprint resulting from stress/shear strain and sediment during the burial process of inclined *Tuzoia* carapaces (see Numerical Model Results). Evidence of this deformation is commonly provided not only by the local absence of trace fossils or presence of highly modified structures, but also by wrinkles, or a visible rough texture of the sediment surface.

Relatively well-preserved *Tuzoia* specimens displaying moderate to high density of trace fossils tend to show the highest ichnodiversity (figure 5*c*,*d*). In contrast, poorly-preserved *Tuzoia* displaying high density of structures typically show low ichnodiversity, although the size range of structures can be very wide (figures 2 and 5*e*,*f*). Identification of structures depends strongly on preservation. Further, compaction can result in superposition of structures that may have been produced at slightly different levels within the sediment during one colonization episode, or at different times (i.e. palimpsest surface). As a general observation, larger structures (width greater than 1.2 mm) tend to display more compressed cross sections, whereas smaller structures (width less than 0.8 mm) are commonly more circular to slightly only compressed in cross section. As previously discussed, pervasive iron staining and compression can strongly distort the morphology of structures to the point of precluding meaningful ichnotaxonomic assessment. Elemental mapping of surfaces of a selected bioturbated *Tuzoia* sp. (figure 2*i*,*j* and electronic supplementary material, figure S2) fails to reveal any significant difference in mineralogy between trace fossils and the hosting sediment. Iron enrichment is interpreted as reflecting late diagenetic and weathering products. Accordingly, no clear mineralogical evidence of early mineralization can be provided at this point. However, some trace fossils provide strong evidence for the absence of severe compaction (e.g. pellet-infilled structures, micrometric *Pilichnus*), unquestionably supporting early mineralization and hardening of biogenic structures compatible with their three-dimensional preservation (albeit with strong variations in compression rates). Additionally, porosity retained in the sediment confined between the valves (see Numerical Model Results) could have provided appropriate conditions for cementation during the compaction process.

## 5. Numerical model results

**Figure 7.**
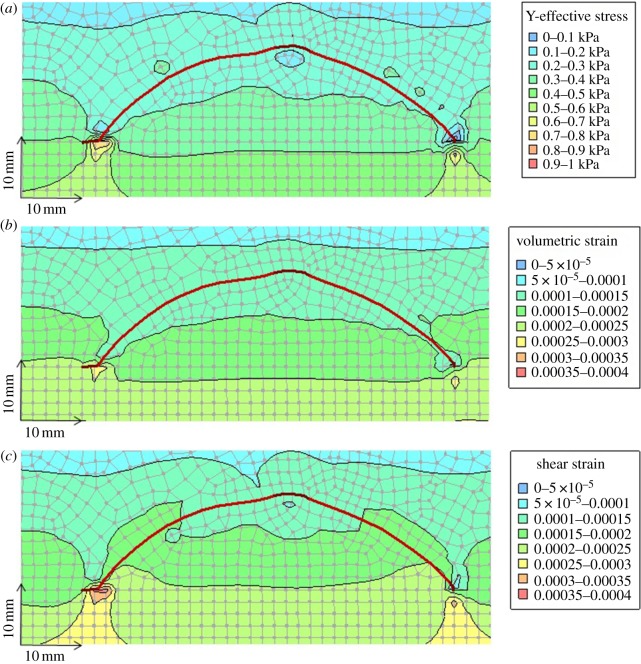
Cross-sectional view of *Tuzoia* carapace model geometry (see also figure 1). (*a*) Vertical effective stress surrounding carapace at 40 mm burial depth (measured at the margins of the carapace). (*b*) Volumetric strain surrounding carapace at 40 mm burial depth. (*c*) Shear strain surrounding carapace at 40 mm burial depth. Note that model outputs identifying sediments which have yielded (i.e. where sediment strength has been exceeded, resulting in plastic deformation of non-lithified sediments) are not present in this figure, as no yielding has occurred in this scenario.

The *Tuzoia* model was run under different burial conditions in order to assess the role of stress/shear strain on carapace deformation features and trace-fossil distribution and morphology, and to make inferences on preservational potential (see input parameters used in electronic supplementary material, numerical model). Model outputs generated for 0.04 m (40 mm) burial depth at the margins of the carapace (which corresponds to approximately 0.025 m burial depth at the peak of the carapace) are shown in figure 7*a–c*. Figure 7*a* shows that stresses due to the overlying sediments are concentrated under both edges of the carapace. The carapace is essentially serving as an arch, shielding the sediments underlying the central part of the carapace from stress by transmitting stresses to the sediment supporting the edges of the carapace. At 0.04 m burial depth, although stresses have become slightly elevated beneath the carapace edges, at no point in the model domain has sediment strength been exceeded (i.e. yielding and plastic deformation of the sediments has not been initiated). However, as shown in figure 7*b*,*c*, elastic deformations resulting from increased stresses have resulted in compression (volumetric strain) and distortion (shear strain) of the sediments near the carapace, with strain maxima occurring beneath the edges of the carapace.

**Figure 8.**
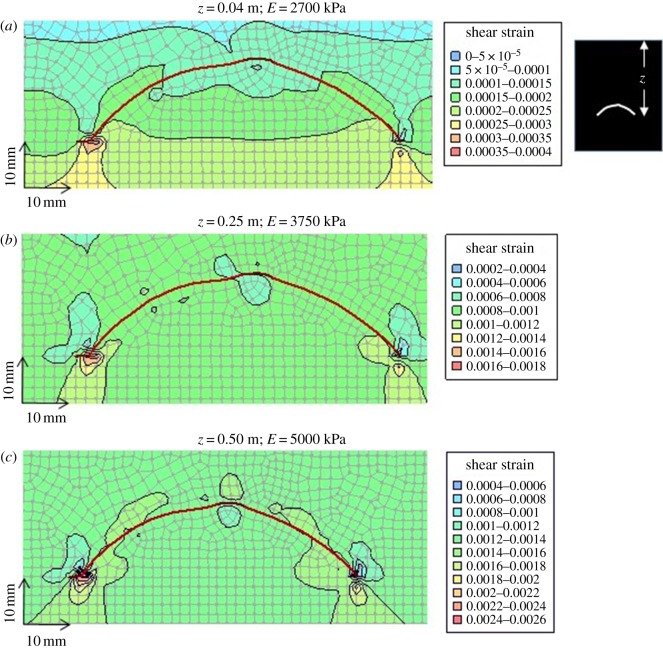
Cross-sectional view of *Tuzoia* carapace model geometry (see also figures 1 and 7). Progressive development of shear strains in sediments surrounding a horizontally-orientated carapace with increasing burial depths. (*a*) At 0.04 m burial depth, shear strain maxima are limited to small areas in the sediments underlying the left and right margins of the carapace. (*b*) At 0.25 m burial depth, the zones of relatively high shear strain noted in (*a*) have grown, and zones of elevated shear strain have initiated in the sediments above and to either side of the lateral ridge (peak) of the carapace. (*c*) At 0.5 m burial depth, the patterns of shear strain are similar to those observed in (*b*), though they have roughly doubled in magnitude. Notable in all three images is the fact that a zone of low shear strain (i.e. undisturbed sediments) is predicted in the sediments beneath much of the carapace (from the lateral ridge (peak) to the inner edges of the afore-noted shear strain maxima beneath the carapace margins), and above both margins of the carapace.

Figure 8*a–c* shows the effects of increasing burial depth (0.04 m, 0.25 m and 0.50 m, respectively) on the geomechanical response of sediments surrounding the carapace. For simplicity, only shear strains are shown in these outputs because the general pattern of vertical effective stress (see ‘Y-Effective Stress’ in figure 7*a*) is similar to the patterns of volumetric and shear strain (figure 7*b*,*c*). The results show that strain (and, by association, stress) magnitudes increase with depth, and that the maximum concentration of strain occurs beneath the edges of the carapace for all depths considered. The results also show that zones of increased strain (hence stress) develop above the carapace, both above the lateral ridge (central peak) and along both flanks (i.e. between the ridge and both margins). Conversely, areas of low strain (hence stress) are particularly well developed in the region beneath the lateral ridge and beneath most of the carapace at shallow burial depths (figure 8*a*), and in a restricted region immediately above the strain maxima all through the burial process modelled (figure 8*a*–*c*).

Based on recent observations in marine muds [13,60,61] and also inferences based on penetration depth recorded in Burgess Shale deposits, we assume that the bulk of the meiofauna and small macrofauna were able to colonize and actively bioturbate the uppermost 2.0–2.5 cm of sediment. Biogenic structures could have been produced below or above carapaces shallowly buried in sediment gravity flow deposits or partially exposed at the sediment surface. In fact, compaction resulting in the decrease of pore space is a determinant parameter of meiofaunal distribution in muddy bottoms [13]. The model results suggest, however, that geomechanical processes are unlikely to have influenced organisms' ability to produce traces due to the very low strains and stresses generated at shallow burial depths (i.e. the first few centimetres). However, as burial depths increased well beyond the maximum depth of burrowing penetration, localized stress maxima (or minima) around the carapace likely began to preferentially destroy (or preserve) traces. Trace-fossil assemblages showing a preferential distribution under the lateral ridge area (e.g. figure 5*a*,*b*) could thus, in principle, be explained in terms of taphonomic overprint as these regions are predicted to have low strains (hence stresses) below a buried carapace affected by increasing compaction. Long structures following the margin (3–5% bioturbation density) could record structures formed above the carapace (cf. figure 8), whereas evenly distributed trace fossils confined within the perimeter of the carapace could mostly be a result of the relatively low stress conditions below the carapace.

**Figure 9.**
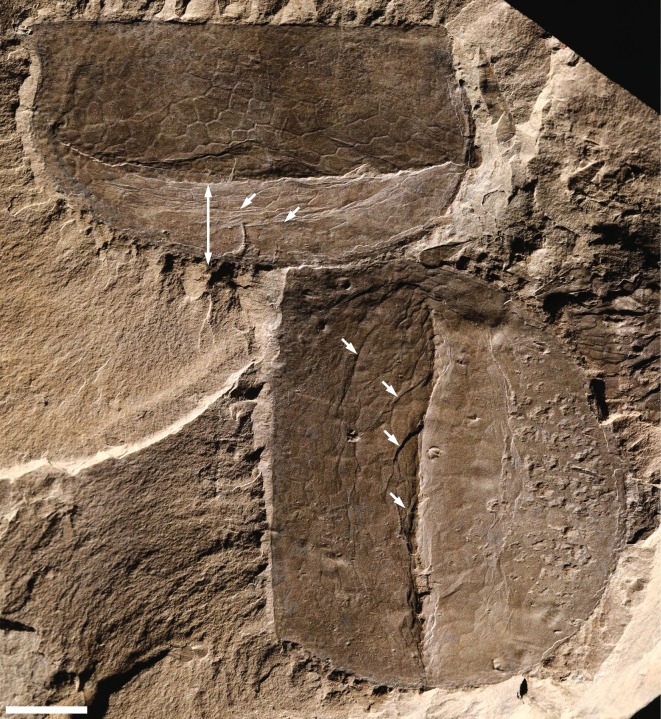
Specimens showing asymmetric (upper left) and relatively symmetric (right) collapse, and evidence of carapace failure features (i.e. wrinkles). ROMIP 57522, *Tuzoia* Bed, Fossil Ridge. Scale bar is 1 cm.

**Figure 10.**
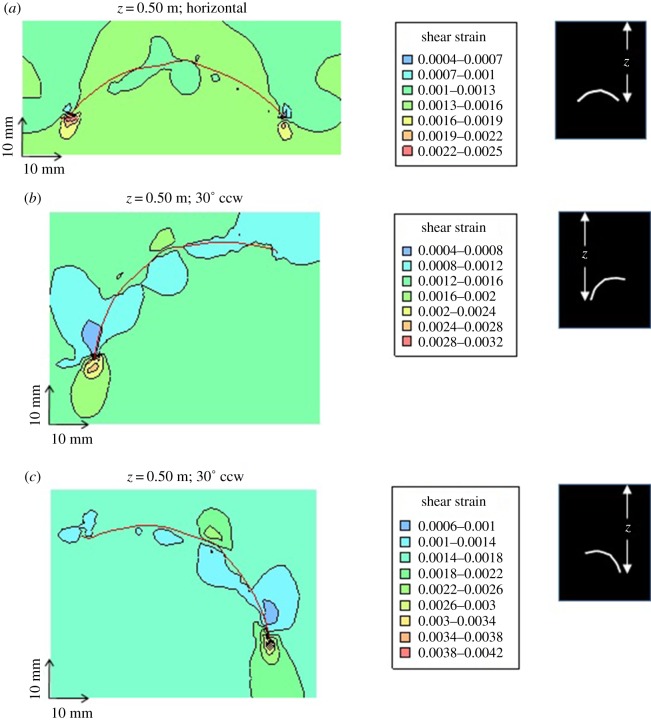
Cross-sectional view of *Tuzoia* carapace model geometry (see also figures 1, 7 and 8). Comparison of shear strain distributions for different carapace orientations, at 0.5 m burial depth. (*a*) Horizontally-orientated carapace (reformatted version of results shown in figure 8*c*). Shear strain distribution is approximately symmetrical, with peak values occurring beneath both margins of the carapace. (*b*) 30° ccw orientation, showing asymmetrical shear strain distribution with peak values occurring beneath the dorsal (left) margin. (*c*) 30° cw orientation, showing asymmetrical shear strain distribution with peak values occurring beneath the ventral (right) margin. Notable in all three images is the fact that a zone of low shear strain (i.e. undisturbed sediments) is predicted beneath the lateral ridge (peak) of the carapace.

Numerous *Tuzoia* specimens show compaction features indicating that they were not completely horizontal at the time of entombment; that is, they display clear asymmetry in the distribution of deformation features. We therefore decided to conduct additional analyses to assess the geomechanical effects on a tilted carapace (i.e. one which was deposited in an inclined position). This idea is compatible with *Tuzoia* carapaces that were transported by currents [30] or incorporated into obrution flows [15] rather than having passively settled from the water column onto the sea bottom (see *the colonization window*). Several studies have demonstrated the pliability of non-biomineralized arthropod carapaces, both in fossil and extant forms (e.g. [62] in naroiids; [63] in xiphosurids). However, the specific range of conditions for plastic (i.e. pliable) and brittle deformation are not well understood for a vast number of fossil arthropod groups. Based on the distribution of features such as wrinkles (figure 9), it is reasonable to hypothesize that many carapaces were buried at an angle to the bedding plane (i.e. were buried tilted, in a non-horizontal position). Analyses were conducted for tilt angles of 30° counterclockwise (ccw) and 30° clockwise (cw), for burial depths of 0.5 m. As expected, the results for the horizontal scenario (figure 10*a*) are approximately symmetrical; subtle asymmetry results from asymmetry in the carapace shape either side of the lateral ridge, and the different shape and increased thickness of the dorsal (left) margin of the carapace compared to the ventral (right) margin. The strain (stress) distributions for the 30° ccw scenario (figure 10*b*) are highly asymmetrical, with the strain (stress) maximum occurring beneath the left (deepest) margin of the carapace, but low strains (stresses) occurring beneath the right (uppermost) margin. Strains (stresses) are relatively high above the lateral ridge, and relative low and expanded below the lateral ridge and in a wide area immediately below the carapace. Interestingly, a low strain area is particularly evident above the left margin of the carapace, suggesting potential preservability of some traces produced above the carapace. The strain (stress) distribution for the 30° cw scenario (figure 10*c*) is similar to the 30° ccw scenario, though rotated in the opposite direction. Considering several carapaces buried at several orientations, one consistent expectation would be good trace preservation (due to low strains) below the lateral ridge, and in a relatively broad region immediately below the internal carapace surface, and poor preservation above the ridge and below at least one margin (i.e. lowermost margin in tilted positions).

**Figure 11.**
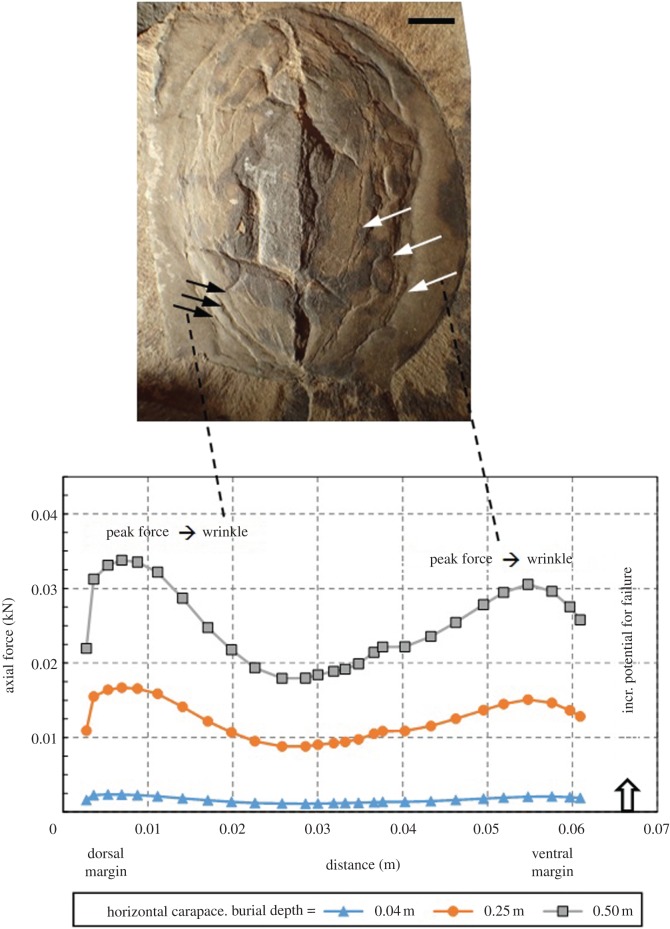
ROMIP 57396. Predicted axial forces within a horizontal carapace, for burial depths of 0.04, 0.25 and 0.5 m. The *x*-axis represents horizontal distance from the dorsal margin of the carapace (which is the left margin for the specimen shown), extending left to right along a line of section positioned at mid-length along the margin (similar to line X-Y in figure 13). Axial forces are compressive along the entire carapace, with magnitudes ranging from a few thousandths to a few hundredths of a kilonewton at burial depths of 0.04 to 0.5 m, respectively. Given that axial forces will ultimately cause compressive failure of the carapace, a nearly symmetrical failure sequence is suggested, initiating near both margins of the carapace, where axial forces reach their largest amplitudes. Scale bar is 1 cm.

**Figure 12.**
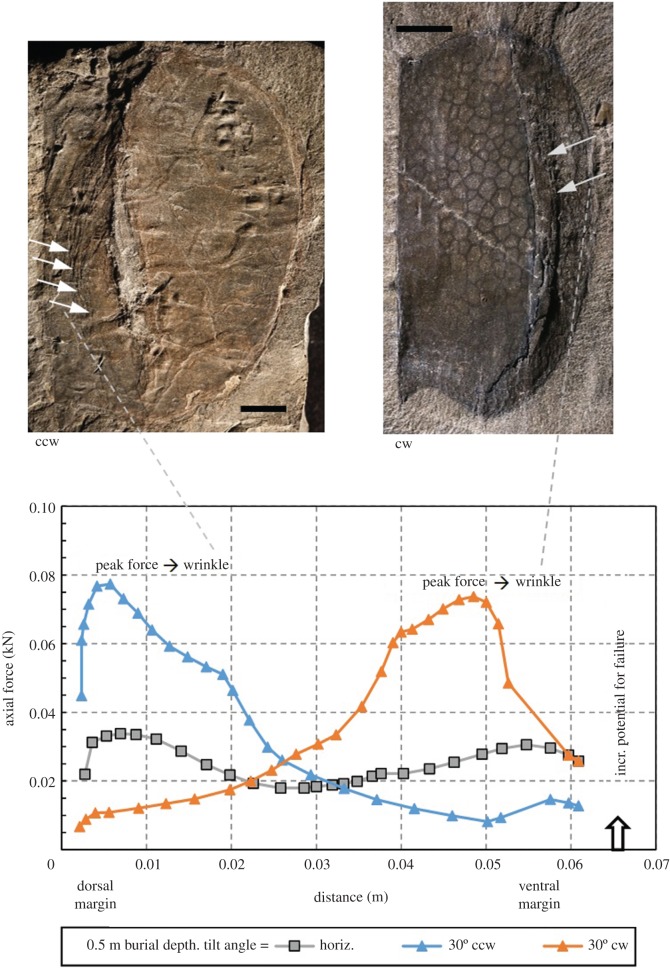
ROMIP 57409 (left) and ROMIP 64581 (right). Predicted axial force within carapace at 0.5 m burial depth for tilt angles of 30° ccw and 30° cw. The *x*-axis represents horizontal distance from the dorsal margin of the carapace (which is the left margin for both specimens shown), extending left to right along a line of section positioned at mid-length along the margin (similar to line X-Y in figure 13). Axial force distribution becomes highly anisotropic for the tilted scenarios, with peak amplitude occurring near the deepest margin of the carapace (i.e. near the dorsal margin for the 30° ccw scenario, and near the ventral margin for the 30° cw scenario). This suggests that carapace failure would occur preferentially near the dorsal margin for the 30° ccw scenario, and near the ventral margin for the 30° cw scenario. Scale bars are 1 cm.

In addition to analysing sediment stresses and strains, the model addresses the axial forces that develop below the carapace during burial. For horizontal carapaces, axial forces are compressive along the entire length of the carapace, with magnitudes ranging from a few thousandths of a kN at 0.04 m burial depth to a few hundredths of a kN at 0.5 m burial depth (figure 11). The distribution of these forces is approximately symmetrical, reaching peak values towards the dorsal and ventral margins of the carapace, where its radius of curvature is smallest. As demonstrated in figure 12, which shows model results for three scenarios at 0.5 m burial depth, axial forces within the carapace become highly asymmetrical for tilted carapace scenarios. In both tilted scenarios, axial forces reach their peak towards the deeper margin of the carapace; i.e. towards the dorsal (left) margin for the 30° ccw scenario, and towards the ventral (right) margin for the 30° cw scenario. Also, the peak magnitudes reached for the tilted scenarios (between 0.07 and 0.08 kN) are roughly twice as large as the peak values modelled for the horizontal carapace. Two specimens are shown in this figure, to demonstrate the connection between asymmetry predicted by the model and that observed in compacted carapaces. The images for both specimens are oriented with the dorsal margin on the left, and the ventral margin on the right. The image on the top left is consistent with model results for the 30° ccw scenario, because wrinkling has occurred preferentially between the dorsal margin and the lateral ridge. The image on the top right is consistent with model results for the 30° cw scenario, because wrinkling has occurred preferentially between the lateral ridge and the ventral margin.

The locations and magnitudes of peak compression within the carapace are important because they provide insight into the processes resulting in failure (and ultimately flattening) of the carapace. Given that the axial forces within the carapace are compressive (figures 11 and 12), the general failure mode will be compressive. Depending on the ductility of the carapace at the time of failure, this could manifest itself as rupture (brittle scenario) or buckling (ductile scenario). Though evidence for both of these processes is present in the specimens investigated, buckling (manifested by wrinkles observed in the carapace) is far more common. Rupture is rare and originated later. Although insufficient information is available to support a numerical analysis of compressive failure of the carapace, it is reasonable to assume that buckling is likely to initiate when the sediments adjacent to the carapace begin to yield (i.e. compress more rapidly due to the onset of plastic deformation). In other words, prior to yielding (and associated plastic deformations), the carapace is supported on both faces by unyielded sediment which restricts its capacity to deform preferentially in either direction. Once the sediment has yielded, the support on opposing faces of the carapace is likely to become more asymmetrical, hence promoting buckling of the carapace. This criterion underlies the selection of 0.5 m as the maximum burial depth considered in the scenarios discussed above, because this is the approximate depth at which sediment yielding initiated. We suggest that the zones of peak strain and stress concentrations identified prior to buckling will initially become zones of maximum plastic deformation upon yielding, though the locations of these maxima will migrate with time due to progressive buckling of the carapace and associated stress redistribution. Assuming a sedimentation rate of 10 cm per 1000 years [64], which is a typical sedimentation rate in distal settings, such as is hypothesized for the Great Phyllopod Bed, it would have taken roughly 5000 years to deposit 0.5 m of sediment. These estimations are consistent with early mineralization of the trace fossils well before severe compression of the carapaces.

**Figure 13.**
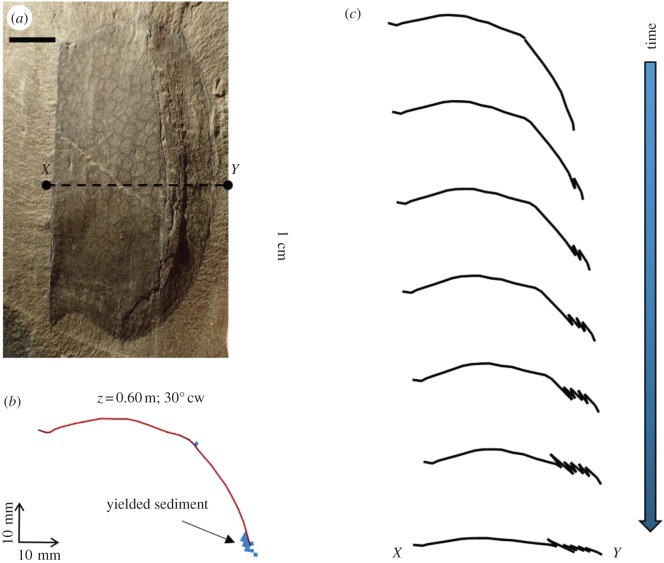
(*a*) *Tuzoia* carapace illustrating progressive asymmetric compaction. ROMIP 64581, *Tuzoia* Bed, Fossil Ridge. This sample shows features analogous to the 30° cw scenario modelled in this work—see figures 8*c* and 11. Note that the area between the lateral ridge and ventral margin has been preferentially shortened. Wrinkles are visible in this area, oriented sub-parallel to the ventral margin. (*b*) Location of yielded sediments (blue shading) predicted for the 30° cw scenario at 0.6 m burial depth. Buckling of the carapace is expected to initiate near the ventral margin, where axial forces in the carapace are high and sediment yielding has occurred. (*c*) Conceptual representation of the manner in which wrinkles, shortening and flattening of a tilted carapace would occur during the burial process. Scale bar is 1 cm.

The results of our modelling also allow us to hypothesize and provide a schematic of progressive, anisotropic formation of wrinkles that map onto the actual features observed in several *Tuzoia* carapaces exhibiting deformation features of the type shown in figure 13*a*. This specimen is an analogue for the 30° cw scenario shown in figures 10*c* and 11. As shown in figure 11, peak axial force (hence the greatest potential for buckling) is predicted near the ventral margin of the carapace. As shown in figure 13*b*, sediment yielding is predicted near this margin as the burial depth exceeds 0.5 m. As such, buckling of the carapace would be expected to initiate near the ventral margin. Figure 13*c* illustrates the authors' conceptual model of progressive failure of the carapace with increased burial. This result is consistent with the fact that asymmetric deformation is a common feature in many unbioturbated and bioturbated carapaces displaying preferential distribution (figure 5*e–h*). In many of these specimens, preferential shortening is a common feature, either in the region above the lateral ridge (i.e. between the hinge line (dorsal margin) and the lateral ridge, similar to the 30° ccw scenario in figure 10*b*) or below the lateral ridge (i.e. between the lateral ridge and the ventral margin, figures 5*e*,*f* and 13*a*). Abnormal trace-fossil morphologies (e.g. drastically changing width along its path) and their virtual absence in predicted zones of strong deformation indicate that these *Tuzoia* specimens were buried in a tilted orientation. At this point, it is worth revisiting one of the assumptions underlying the numerical modelling, i.e. that carapace mechanical properties remain constant over time. This is actually a valid assumption for the majority of the studied material. However, allowing for the fact that degradation processes acting on the carapace may have weakened some long-exposed specimens over time, the failure geometry illustrated in figure 13*c* may have developed at a lesser burial depth than predicted by the models used in this work.

## 6. Discussion

### 6.1. Two end-member hypotheses: the *Tuzoia* garden versus the shielding carapace

There are two end-member scenarios envisioned to explain the occurrence of trace fossils associated with non-biomineralized carapaces, emphasizing either predominantly ecological controls at one end (the *Tuzoia* garden hypothesis) or determinant taphonomic controls (the shielding carapace hypothesis) at the other. In the *Tuzoia* garden, carapaces (moulds or consumed soft tissue carcasses) created opportunity for ecologic interactions for a wide size range of benthic organisms (i.e. micro, meiofauna and small macrofauna). If most *Tuzoia* specimens were carcasses, C-rich soft tissues would have supplied a rich food source for benthic organisms, particularly scavengers (see [25] on the presence of *Myoscolex* associated to *T. australis*). This scenario would favour *Tuzoia* as a nektobenthic arthropod, being frequently trapped in mass flow events. However, this scenario is not supported by fossil evidence which points to a pelagic mode of life ([15], but see [25]). In fact, only a couple of *Tuzoia* among hundreds of specimens show any signs of soft tissues, rather than the most recalcitrant carapaces [15,16]. Thus, in the Burgess Shale, *Tuzoia* carapaces most likely represent moults (or already fully decayed specimens) which may have settled at the bottom before being remobilized by frequent mass flow currents to be finally re-deposited, most likely in a nearby environment. In this ecological scenario, carapaces do not provide a direct source of labile organic matter, instead they act as structural elements that engineered the distribution of resources in the environment, creating surfaces for bacterial growth (cf. [65], fig. 4*D*,*E*) and reinforcing bottom-up links and small loops in the trophic web [13]. As an additional advantage, confinement below the carapace may have offered convenient protection against predators.

In addition, the presence of the carapace at the sediment–water interface or shallowly buried in the sediment would have increased the geochemical gradient between the muddy oxygen deficient sediment and the water column, further favouring the growth of diverse populations of bacteria and, in turn, attracting bacteria-grazing organisms [4]. Bacterial spatial fluctuations (e.g. preferentially concentrated on an organic surface) influence the distribution of microfauna and the abundance of meiofauna, which in turn have complex interactions with small macrofauna. Interactions between bacteria and meiofauna are diverse and still understudied [13]. Nitrogen- and Phosphorous-containing metabolites of meiofauna foster the growth of bacteria and excreted mucus enhance both the growth of bacteria and colonization by microorganisms, which are cropped by meiofauna and small macrofauna. Morphological features of some trails and burrows studied, such as simultaneous branching and secondary successive branching, imply re-visitation of structures and suggest bacterial grazing, whereas actively infilled structures (e.g. pellet-infilled and annulated burrows) record the activity of deposit feeders, which probably also benefited from augmented bacterial populations growing on and, particularly, below carapace surfaces.

In modern settings, meiofauna and small macrofauna have been identified as important links in the trophic web, providing substantial food to higher trophic levels [60,66–69]. This transfer of meiofaunal energy to higher levels is particularly significant in muddy sediments where meiofauna (both permanent and temporary) is concentrated in surficial levels and is easily accessible to numerous macrofaunal predators [13]. The Burgess Shale biota at the Walcott Quarry is dominated by nektobenthic arthropods that could have possibly fed on microfaunal and meiofaunal components as part of their diet; for example, the small arthropod *Yohoia* has been suggested to be a micropredator [70]. In addition, exceptionally preserved trackways from the Kicking Horse Member reveal that large size tegopeltid arthropods were fast predators skimming on the sediment surface and probably preying on small benthic macrofauna and meiofauna [6]. There is also direct evidence from gut content analyses that some predators or scavengers, such as the priapulid worm *Ottoia* and the arthropod *Sidneyia*, preyed on small benthic fauna as well [71,72]. In this ecologic scenario, carapaces can be considered physical structural elements engineering energy transfer in Cambrian food webs [73]. Although able to move around on and into the surrounding sediment, the mobile epifauna and shallow infauna (i.e. micro, meiobenthos and small macrobenthos) may have preferentially concentrated around bacterially enriched carapaces and rare carcasses (providing labile soft tissue to scavengers), functioning as attractors of biogenic activity. In short, the *Tuzoia* garden hypothesis entails the idea that trace fossils occur in lower density and are fundamentally less diverse in the surrounding sediment than trace fossils directly associated with carapaces. Ecological factors have often been invoked in the literature to explain trace fossil–body fossil associations in numerous Cambrian Burgess Shale-type deposits (e.g. [2,4,5,74]).

At the other end of the spectrum, the association of trace fossils with non-biomineralized carapaces could be explained entirely as the result of a taphonomic overprint, essentially denying any ecological control. In this view, even dense clusterings of biogenic structures would not necessarily require invoking ecological interactions: instead the fortuitous spatial proximity, mechanical properties of the flexible cuticle and compaction history would provide the necessary conditions to explain all documented trace fossil–carapace associations. According to this scenario, the carapace serves as a ‘shield’ mediating compaction of biogenic structures, essentially controlling and delaying yielding and the effects of stress. The recorded differences in trace-fossil density and diversity between the carapace and the host rock are then explained as the result of taphonomy. Higher stress/shear and compaction rates affecting biogenic structures in the host sediment may have caused the total destruction of many morphological types and the extreme morphological modification of those structures that survived compaction (e.g. strongly flattened, discontinuous burrows in the surrounding sediment). This hypothesis entails a widespread, surficial to shallow-tier community of small benthic organisms—mostly grazers and deposit feeders inhabiting a microbially stabilized, organic-rich muddy sea bottom. This scenario is consistent with the fact that Burgess Shale distal deposits formed under fluctuating, but dominantly dysoxic conditions, and lack a well-developed mixed layer and tiered infaunal communities, therefore allowing for preferential preservation of surficial and shallow-tier trace fossils [75]. According to this hypothesis the apparent patchiness in the distribution of biogenic structures results from increased strength of biogenic structures mostly, but possibly not exclusively, below the carapace due to delayed burial, and differential compaction enabled by the local presence of non-biomineralized cuticle.

### 6.2. Carapaces as promoters of biogenic activity and mediators in trace-fossil preservation

It is clear from the study of biogenic structures associated with *Tuzoia* that a very limited number of trace fossils can be traced in and out of the area of the carapace. As previously outlined, biogenic structures that cross the carapace boundary into the surrounding sediment drastically change in morphology from conspicuous, positive reliefs (‘3D preservation’) with sharp, well-defined boundaries to very compressed, flat structures with diffuse boundaries (‘2D preservation’). Accordingly, morphological evidence strongly supports the idea that the non-biomineralized carapace did play a significant role as a mediating agent in crossing the fossilization barrier. Based on this evidence, and adding the results from stress/shear strain modelling, low density-low ichnodiversity assemblages, particularly those in which simple trails or burrows cross through the carapace, may be most parsimoniously explained by coincidental association. This interpretation applies not only to the Burgess Shale material analysed here, but also to many illustrated trace fossil–body fossil associations in other Burgess Shale-type deposits (e.g. [20,76]). However, those trace fossil–body fossil associations that display relatively high densities of trace fossils and involve higher ichnodiversity or show particular morphologic patterns recording confinement (e.g. circling and overcrossing associated with *Pararotadiscus*, see fig. 5 in [1]) challenge the strictly taphonomic scenario.

Results of the numerical model analysis clearly suggest that: (i) The carapace can shield underlying sediment and structures from mechanical stress for a finite time before deformation and strong compaction. In principle, this delay could have provided enough time for early diagenetic processes, such as early cementation of infills or wall structures, to take place (cf. [77,78]), although this remains a hypothesis to further explore and as yet is not confirmed by our elemental maps (see electronic supplementary material, figure S2). In particular, enhanced conditions for early diagenetic processes are expected in the less-compacted, porous, microbial-rich, reducing sediments shielded by the carapace. An increased geochemical gradient due to the sealing effect of the organic cuticle most likely resulted in decreased oxygen diffusion into the sediment and optimal conditions for early cementation beneath the carapace [79,80]. (ii) There are critical areas of predicted high stress/shear strain at carapace margin both along the dorsal and ventral margins, and upper surface of the valve in a horizontally compacted carapace (figure 8), and an area of low stress (shear strain) in the central area underlying the carapace (stress minimum beneath the lateral ridge). Some trace-fossil distribution patterns can be successfully interpreted based on this model (e.g. figure 5*a*,*b* and *e*,*f*). Inclined carapaces present more complex compactional scenarios, suggesting the potential preservability for structures on some localized areas on top of the upper valve (figure 10*a*,*b*). In short, the effect of the carapace as a medium promoting preservation is supported by both numerical modelling and the numerous *Tuzoia* specimens that exhibit deformational features and trace-fossil distributions compatible with the predicted stress (shear strain) distribution model. In other words, the shielding carapace hypothesis may parsimoniously explain trace-fossil assemblages comprising a localized concentration of biogenic structures coincident with stress minimum, or localized absence of trace fossils and presence of abundant wrinkles in areas of predicted high stress (shear strain). The taphonomic model, however, is clearly not a satisfactory explanation for some more diverse and morphologically more complex trace fossil–carapace associations studied.

Fundamental theory on stress analysis around tunnels suggests that cylindrical structures (i.e. vermiform-like) in homogeneous sediment should display similar compaction pathway regardless of size (i.e. both small and large diameter cylindrical structures would being flattened at a similar rate). However, it is observed that morphological differences within an assemblage (e.g. differences in compression, sharpness of structure boundaries) do occur (e.g. some of the larger burrows are extensively flattened). It is suggested that these may have resulted from biomechanical factors not accounted for in conventional stress analysis (e.g. the effects on strength and deformability of mucus reinforcement or active infilling). These morphologic differences are interpreted as reflecting constructional differences most likely linked to the palaeobiological affinity and ethology of the producer. In fact, specimens displaying relatively high densities of trace fossils (11–25% bioturbated area) that do not record asymmetric deformation (i.e. buried in an inclined position) display an even distribution of structures that suggests that final observed distribution may have been controlled primarily by the physical presence of the carapace and the advantages that it provided, and secondarily by the effects of compression stress. Long, continuous structures are rare, but where present, they tend to follow the ventral, anterior or posterior margin of the carapace, or move parallel to the hinge, recognizing the physical presence of the carapace (figures 4*a*,*b* and 5*c*,*d*)*.* Whether these trace fossils observed near the carapace perimeter (in the absence of inflections in the carapace margins) formed in the underlying sediments, slightly inwards of the shear strain maxima, or in the zones of low shear stress overlying the margins, is a compelling question. Pellet-infilled structures, delicate bifurcating structures and interconnected burrows are restricted to carapaces. Although tiny, delicate bifurcating structures can be susceptible to destruction during the compaction process, pellet-infilled structures are resistant to compaction and are well known in the stratigraphic record (e.g. [44,81]). Pellet accumulations are relatively common in association with shelly material and have been related to the activities of scavengers in fossil trilobites, molluscs and echinoderm tests, suggesting an ecological relation [82]. *Alcyonidiopsis* and other pellet-infilled structures, such as *Tubotomaculum* [81] and *Phymatoderma* [83], commonly occur in relatively deep-water mudstone and very fine-grained sandstone, indicating that they have the potential to survive the destructive effects of compaction outside the carapace. In fact, tiny pelletized structures have been recognized in the Burgess Shale ([14], fig. 1.18*H-1*) and in Chengjiang ([2], fig. 3).

### 6.3. The colonization window

The occurrence of trace fossils associated with non-biomineralized carapaces can be analysed from different perspectives and at different scales. At the scale of the carapace itself and based on the evaluation of the density and diversity of trace fossils, we have depicted the interplay of taphonomic and ecological factors. In order to detect major variations at the stratigraphic scale, however, the association of trace fossils and non-biomineralized carapaces is explored by comparing occurrences in the three Burgess Shale localities analysed. One of the questions we attempt to address is why trace-fossil densities are so variable among *Tuzoia* populations from different sites.

**Figure 14.**
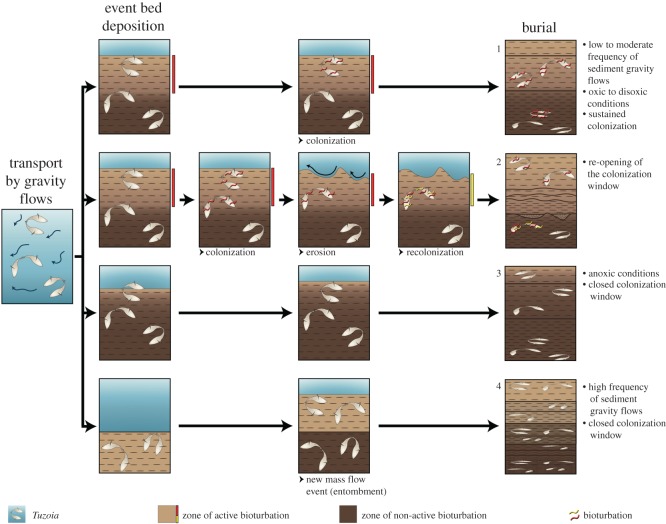
Taphonomic pathways for trace fossils-*Tuzoia* associations in Burgess Shale deposits of the Canadian Rockies. Transport by a mass flow is modelled as the main depositional mechanism. In this scenario carapaces may have been transported from a nearby location or from a more proximal position (pre-slide environment *sensu* [85]. Under oxygenated conditions, a thin zone of active bioturbation occurred above a zone lacking active bioturbation (the historic layer). Examples of scenario 1 are found in Stanley Glacier. In this case, sustained colonization windows due to low to moderate frequency of mass flows allowed extensive bioturbation of the carapaces. In addition, the fact that almost all *Tuzoia* specimens studied contain trace fossils indicates that carapaces were emplaced within the zone of active bioturbation. This is consistent with relatively thin event beds. A diverse set of scenarios can be reconstructed from occurrences in the *Tuzoia* bed, Raymond Quarry, essentially exemplifying each of the elucidated pathways. Occurrences illustrated by scenario 2 imply relatively thick, erosionally based event beds and common recolonization after sediment erosion and by-pass (recorded by gutter casts and other erosional features on bedding surfaces). The fact that not all *Tuzoia* specimens found on a single slab (but on different laminae) contain trace fossils suggests that some carapaces were emplaced below the zone of active bioturbation, and that erosion did not reintroduce them to the zone of active bioturbation. Scenarios 3 and 4 represent potential scenarios for Walcott Quarry (4 also for *Tuzoia* bed). In scenario 3, the limiting factor for bioturbation was persistent lack of oxygen. Under anoxic conditions, no zone of active bioturbation was present, whereas in scenario 4 the absence of trace fossils is explained as resulting from high frequency of sediment gravity flows. In either case, the colonization window remained closed. High compaction rates resulted in closely spaced carapaces (although not on the same laminae) that were emplaced at different levels during deposition.

The concept of the colonization window or time available for occupation of the substrate [84] is instrumental to addressing this issue. Carapace colonization might have happened under different scenarios, resulting in contrasting taphonomic pathways (figure 14). First, considering that *Tuzoia* was a pelagic organism that most likely moulted in the water column, moulds are expected to have first arrived at the sediment–water interface via gravity. Carapaces would have resided along the seafloor for some time before becoming entombed by subsequent mud flow deposits involving minimal transport. Bioturbation may have occurred, given the appropriate environmental conditions, before and after burial. However, these biogenic structures are not expected to survive transport. *Tuzoia* carapaces were buried by sediment gravity flows together with a wide range of mostly benthic organisms, some of which were alive and some dead at the time [29,86], with some carapaces and carcasses being bioturbated immediately after deposition (figure 14, scenarios 1 and 2). Erosion of the sediment may in principle have placed previously deeply buried carapaces within the zone of active biogenic reworking, allowing further bioturbation of some exhumed carapaces (figure 14, scenario 2). The absence of trace fossils associated with *Tuzoia* specimens from the mudstones of the Greater Phyllopod Bed in the Walcott Quarry is consistent with observations from carapaces of the other taxa. They too are almost completely devoid of any biogenic activity, with rare exceptions (e.g. *Alcyonidiopsis* on carapaces of *Odaraia*). Although the sporadic presence of trace fossils in mudstone deposits has been mentioned in the literature [30,31,87], traditionally these deposits have been regarded as barren from an ichnological standpoint ([56]; Mángano, personal observations 2009 and 2015). The possible surficial bioturbation illustrated by Powell *et al*. [87] is highly reminiscent of microbially-induced sedimentary structures. However, possible trace fossils were figured by Caron and Jackson ([30], fig. 7*A*). Although it is not clear whether such trace fossils come from the non-laminated mudstone containing soft-bodied fossils or from calcareous siltstone interbeds, they undeniably provide evidence of temporally and spatially localized bioturbation. Caron & Jackson ([30], fig. 7*B*) also illustrated ‘microburrows’, but following closer inspection these small tubular structures probably represent the remnants of organic tubes, possibly of hemichordate worms, such as *Spartobranchus* not trace fossils [88]. One plausible explanation for the rarity of trace fossils in the Walcott Quarry is near complete, highly persistent anoxia at the sediment–water interface, as metazoans cannot survive in the persistent absence of oxygen (figure 14, scenario 3). However, detailed palaeoecological analysis allows for the reconstruction of authochtonous metazoan communities *contra* total anoxia [30]. In addition, geochemical analysis seems to suggest oxygenated bottom waters [87]. A second possibility involves a detrimental role of sedimentation due to high frequency of mass flows and/or high volumes of sediment deposited (figure 14, scenario 4). Sedimentological studies indicate deposition from quasi-sustained density currents that may have resulted in rapid burial [86]. As trace fossils in Burgess Shale deposits are typically restricted to shallow tiers, some events may have resulted in carapace burial well below the reach of bioturbating infauna, particularly in the case of the Walcott Quarry, where event beds are usually thick (up to 10 cm thick after full compaction and dewatering). In addition, high frequency of sediment gravity flows may have precluded colonization of the carapaces. Regardless of the relative roles played by these two factors, namely anoxia and high frequency/volume of episodic sedimentation, the colonization window remained essentially closed during deposition of the Greater Phyllopod Bed.

The uneven distribution of trace fossils in the *Tuzoia* carapaces from the Raymond Quarry Member (including the *Tuzoia* Bed) is consistent with the presence of sparsely distributed burrows, typically associated with shell hash and only very locally occurring in high densities at distinct stratigraphic levels [56]. Many carapaces, however, display complex stress features suggestive of inclined positions during burial. This strongly suggests that the *Tuzoia* Bed records a combination of frequent rapid sedimentation events with high rates of erosion evidenced by gutter casts and other erosional structures [56]. Sediment removal may have made previously buried carapaces available for a subsequent colonization episode by the shallow infauna (figure 14, scenario 2). This may have been the case for those carapaces with high densities of trace fossils. More commonly, frequent episodic events may have allowed *Tuzoia* carapaces to be transported and only briefly available for colonization before being buried beyond the depth of bioturbation (carapaces with low densities of trace fossils).

The overall high densities of biogenic structures in *Tuzoia* from Stanley Glacier suggest an extended colonization window (figure 14, scenario 1). In fact, trace fossils, including trackways, are widespread on many stratigraphic surfaces at Stanley Glacier [3]. Composition and size of trace-fossil assemblages with extensive stratigraphic distribution suggest that bottom conditions were for the most part dysoxic, with oxygen fluctuations reflected by subtle ichnodiversity shifts [3]. Construction of very simple trails, pelletized burrows and galleries involves ecological time on the order of weeks to years, during which skeletons serve as physico-chemical elements in the environment that control biogenic activity for a limited time span. In this scenario, *Tuzoia* must have been available long enough at the sediment–water interface or shallow depths (less than 2.5 cm) in between deposition from gravity flows to promote the development of small benthic communities that concentrated around carapaces and benefited from the carapace protection (i.e. refugia from larger arthropod predators) and niche construction generated by enhanced microbial activity.

## 7. Conclusion

Two main hypotheses are explored in order to understand the significance of trace fossils associated with non-biomineralized carapaces, mostly based on the analysis of *Tuzoia* from several Burgess Shale sites in the Canadian Rockies. The ecological *Tuzoia* garden hypothesis highlights the heterogeneity of Cambrian sea floors and the significance of bacteria, meiofauna and small macrofauna as important links in the Cambrian trophic web, whereas the taphonomic shielding hypothesis relies on differential compaction mediated by the non-biomineralized carapace and disregards ecologic links, interpreting the trace fossil–carapace association as coincidental. A numerical model of the entombment of a *Tuzoia* carapace was constructed in order to evaluate the effects of mechanical strain and stress and related to burial. Numerical model results confirm the idea of the carapace as an organic interphase that shields trace fossils from compaction for a limited time window, enough to allow early cementation to occur, before failing and buckling. Although structures could be formed above and below the carapace, optimal conditions for preservation are predicted for biogenic structures produced in the sediment below the carapace based on a more pronounced geochemical gradient. Low-density, low-diversity trace-fossil assemblages may be most parsimoniously explained by this taphonomic model (also supported by asymmetric deformation and distribution of trace fossils). However, relatively high-density and more diverse trace-fossil assemblages, showing spatial confinement within the perimeter of carapaces suggest that bacterially-enriched carapaces may have attracted micro-, meio- and small macrofauna, therefore revealing the presence of poorly known lower levels of the Cambrian trophic web.

The remarkable variability in trace-fossil densities in the *Tuzoia* populations from the different sites is explained in terms of variation in the duration of the colonization window. Absence or extremely rare low-density trace-fossil assemblages (less than 3%) are associated with rapid rates of burial (high frequency and/or volume of sediment gravity flows) and, to a lesser extent, anoxia (Greater Phyllopod Bed at Walcott Quarry). In contrast, high-density assemblages are interpreted as the result of sustained colonization windows in settings affected by a lower frequency of event deposition (and/or greater time averaging) and recurrent oxygen fluctuations, although most likely within the dysoxic range (e.g. Stanley Glacier). In other cases, sediment removal due to erosion and bypass may have made previously buried carapaces available for subsequent colonization (e.g. Raymond Quarry *Tuzoia* Bed).

## Supplementary Material

Additional trace fossil figure

## Supplementary Material

Additional Tuzoia figure

## Supplementary Material

Numerical model, additional figure and table

## Supplementary Material

Table summarizing information of specimens studied

## Data accessibility

The data supporting this article are available in the electronic supplementary material for this manuscript.
