# Supplementary material for: Trace fossils associated with Burgess Shale non-biomineralized carapaces: bringing taphonomic and ecological controls into focus
Source: R Soc Open Sci. 2019 Jan 16;6(1):172074. doi: 10.1098/rsos.172074 (PMC6366168; doi:10.1098/rsos.172074)
Supplement: Additional trace fossil figure [file rsos172074supp1.pdf]

Electronic Supplementary Material – Figure S1

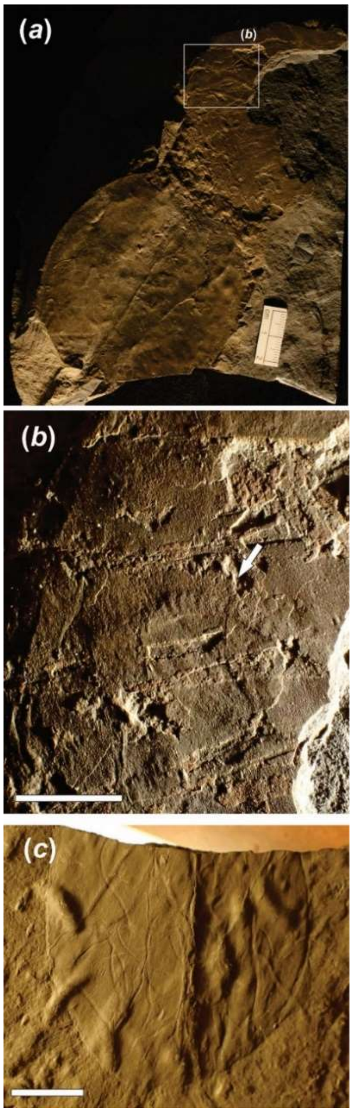

Electronic Supplementary Material Figure 1. Trace fossils associated with *Tuzoia* carapaces. (*a*) General view of two specimens of *Tuzoia* sp.; annulated burrow in fragmented specimen (white box), ROM 64814, *Tuzoia* Bed, Fossil Ridge. (*b*) Close-up of annulated burrow (arrow) shown in *a*. Scale bar is 0.5 cm. (*c*) General view of a high-density trace-fossil assemblage associated with *Tuzoia* sp. Note two clear sets of trace fossils: thin simple trails and burrows mostly confined to the carapace outline and U-shaped burrows clearly visible as positive ridges. These two distinctive set of structures are interpreted as recording a palimpsest assemblage. Although polarity of the bed was not recorded (this is talus material), based on the preservation of U-shaped burrows it is suggested that this surface records a bed sole. Accordingly, U-shaped burrows are overlying the *Tuzoia* carapace and possibly superimposed due to the compactional effect. U-shaped burrows are also present in the host rock. ROM 57533, Walcott Talus, Fossil Ridge. Specimen most likely coming from levels of the Raymond Quarry or higher in the stratigraphic section. Scale bar is 1 cm.
