# Supplementary material for: Trace fossils associated with Burgess Shale non-biomineralized carapaces: bringing taphonomic and ecological controls into focus
Source: R Soc Open Sci. 2019 Jan 16;6(1):172074. doi: 10.1098/rsos.172074 (PMC6366168; doi:10.1098/rsos.172074)
Supplement: Additional Tuzoia figure [file rsos172074supp2.pdf]

Electronic Supplementary Material – Figure S2

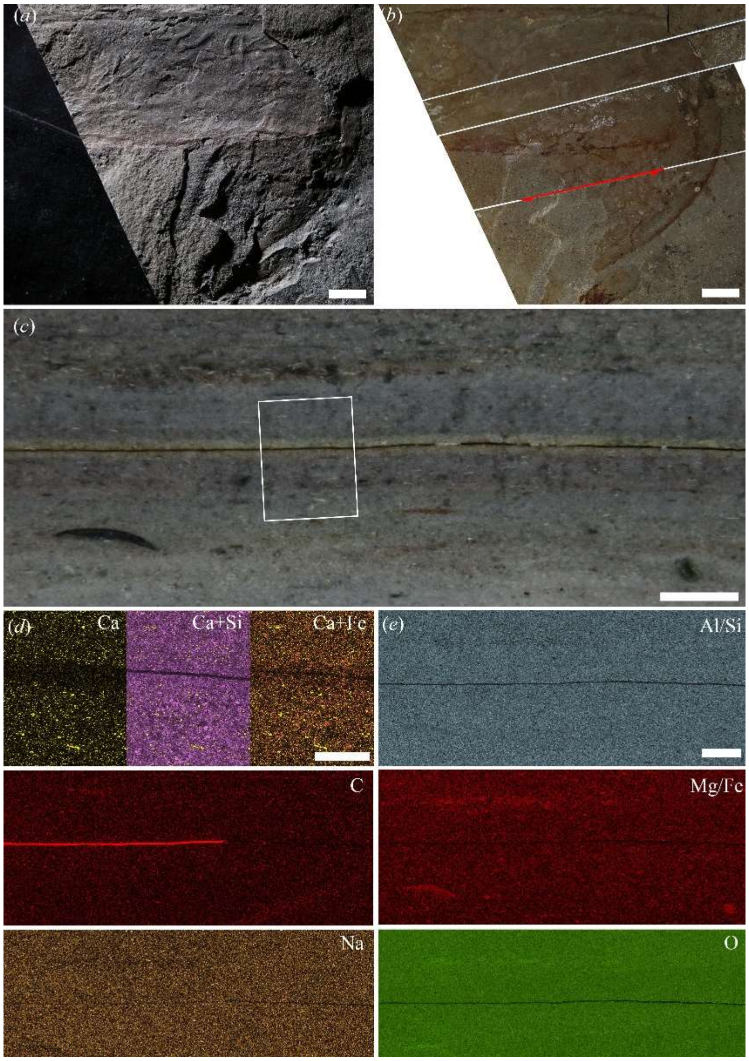

Electronic Supplementary Material Figure 2. Cross sections of a *Tuzoia* carapace. ROM 64825, Stanley Glacier, Kootenay National Park. (*a*) Low angle light emphasizing the presence of burrows. (*b*) Specimens photographed using cross-polarized light after cuts – the line in red represents area mapped. (*c*) Polished cross-section of the part and counterpart. The carapace occurs at the interface along an horizontal yellow zone which likely represents a weathering effect. (*d*) Maps of Ca, Ca+Si and Ca+Fe showing a weathered layer poor in Ca (framed area in c). (*e*) Different elemental maps showing no evidence of variations in mineralogy between the traces fossils and the matrix. The bright carbon zone at the interface between the part and the counterpart is glue holding the two pieces together. The carapace itself is not visible, and the Carbon film has probably weathered out or is too thin to be detected in cross-section. Note that part of the cut probably included both valves, but it is impossible to tell based on cross sections. Scale bars are 1 cm in *(a, b)* 1 mm in *(c)* and 0.5 mm in *(d, e)*.
