# Supplementary material for: Trace fossils associated with Burgess Shale non-biomineralized carapaces: bringing taphonomic and ecological controls into focus
Source: R Soc Open Sci. 2019 Jan 16;6(1):172074. doi: 10.1098/rsos.172074 (PMC6366168; doi:10.1098/rsos.172074)
Supplement: Numerical model, additional figure and table [file rsos172074supp3.pdf]

**Electronic Supplementary Material - Numerical Model**

# Trace fossils associated with Burgess Shale non-biomineralized carapaces: Bringing taphonomic and ecological controls into focus

M. Gabriela Mángano, Christopher David Hawkes, Jean Bernard Caron

## The *Tuzoia* carapace 2-D Model

Although *Tuzoia* is a three-dimensional (3-D) form, we modelled the structure as 2-D providing some assumptions (i.e. model constraints, see below). We consider this 2-D model is able to capture representative patterns of stress and strain during the early stages of burial and compaction.

Assumptions made for the modelling include the following:

1. 2-dimensional (2-D), plane strain geometry. This idealized geometry is commonly used in various branches of solid mechanics, including rock and soil mechanics. Strictly speaking, it is applicable in scenarios where the structure being analyzed has a cross-sectional shape which does not vary with distance normal to the plane of analysis. In such cases, displacements (hence strains) within all points of the structure occur within the plane of analysis, and the values of these displacements do not depend on the distance normal to the plane. 2-D plane strain modelling is common practice even in cases where the underlying assumptions do not, strictly-speaking, hold true. This is largely the case because of the additional expense and effort required to build and run 3-D models, and because the uncertainties associated with model construction (e.g. What – *exactly* – is the 3-D shape of a *Tuzoia* carapace? What – *exactly* – are the mechanical properties of the carapace and surrounding sediments) are likely greater than the degree of inaccuracy incurred by using a simplified 2-D geometry All else being equal, use of the 2-D model leads to under-estimates in the magnitudes of stress and strain concentrations around the carapace, because the out-of-plane curvature (along the length of the carapace) would serve as a broad arch capable of redistributing some of the load generated by overlying sediments. However, the most important arch is the dorso-ventral cross section considered in our model, given its tight radius of curvature relative to the out-of-plane arch. Most importantly, the patterns of stress and strain distributions (i.e. locations of highs and lows) in the cross-section analysed would remain the same for a 2-D versus 3-D model, and these patterns are more important than the magnitudes in this work.

2. Drained loading. This assumption implies that the sediment has sufficient permeability to allow pore water to drain freely out of the sediment as additional load is applied. In the case of this work, the *Tuzoia* carapace is assumed to be impermeable, but the unlithified sediments below, around and above it are assumed to have sufficient permeability to enable drainage of fluids as load accumulates due to burial.
3. Linear elastic – plastic constitutive behavior for sediment. This assumption presumes that sediment deforms in an elastic manner until reaching a critical threshold (peak strength), after which it deforms in a plastic (ductile) manner. In this work, the model is only used up to the point at which plastic deformation of the sediments initiate, as we expect that buckling (wrinkling) of the carapace would be triggered by this plastic deformation, and the model is not capable of accurately simulating wrinkling of the carapace (and associated deformations in the sediment).
4. Linear elastic behavior for the structural elements used to represent the *Tuzoia* carapace. This assumption attempts to simulate the behaviour of retaining structures which are placed on surfaces in order to provide stability. It presumes that the structural elements deform in an elastic manner until reaching a critical threshold, after which they instantaneously lose their capacity to bear load. As noted above, for this work the critical threshold chosen was the onset of sediment yielding, which likely allowed the carapace to buckle due to the compressive forces acting within it. Modelling was terminated after this threshold was reached, because it was deemed impractical to model the complex geometries (e.g. compound series of wrinkles) that would ensue.
5. Carapace has a constant thickness, except for locally thickened zones at the ridge and margins. This condition ignores the polygonal structure of the carapace which most likely reinforces the carapace, potentially modifying some physical properties such as resistance to increasing load.
6. Carapace mechanical properties remain constant over time. This assumption neglects the fact that biological and chemical processes will degrade the stiffness and strength of the carapace over time. Though the long-term load-bearing capacity of the carapace will be over-estimated as a consequence of this assumption, it is the authors' belief that mechanical property degradation may be modest over the time frame of early burial, when most to the interesting processes regarding animal colonization and production of trace fossil occurred, given the refractory nature of chitin.
7. The numerical model developed for this work cannot rigorously account for buckling, nor for plastic deformation of the carapace, hence its results are not considered reliable at burial depths greater than roughly 0.5 m.

Table *1* lists the input parameters used for the model, including comments regarding the rationale for these values. Figure 1 shows the results of sensitivity analysis, which was undertaken to confirm that the general conclusions derived from the model (which stem from the patterns of stress and strain around the carapace) hold true even when accounting for some uncertainty in

the values of the model input parameters. In particular, figures *1b* and *1c* show that the distribution of strain is nearly identical to the base case, even when increasing the sediment stiffness by a factor of roughly four or increasing the carapace thickness by a factor of three, respectively. While it is true that the strain magnitudes change, the locations of zones of high and low strain (hence stress) remain the same. Figure *1d* represents the greatest departure from the base case. This scenario assumes that the carapace did not fill entirely with sediment during burial (which may also be regarded as similar to a scenario in which the carapace did infill, though the sediment within was more porous, hence much softer than the surrounding sediment). For this scenario the general distribution is similar to the base case, though the zones of greatest strain near each margin have grown upwards and inwards (towards to edge of the unfilled cavity), and large zones of low strain exist both above and below the lateral ridge (central peak) of the carapace. What remains true in all scenario is the existence of a zone beneath the lateral ridge which is shielded from high strain (hence stress), in which trace fossils would be preferentially preserved.

Table 1: Parameters used for geomechanical modelling of carapace deformation

| **Parameter** | **Value** | **Comment / Source** |
|---|---|---|
| Young's modulus of sediment surrounding carapace:<br>• At 0 m burial depth<br>• At 0.5 m burial depth | <br><br>2.5MPa<br>5.0 MPa | Representative of stiff clay.<br><br>Value is assumed to increase linearly with burial depth. |
| Poisson's ration of sediment | 0.33 | Representative of stiff clay |
| Cohesion of sediment:<br>• At 0 m burial depth<br>• At 0.5 m burial depth | <br>2.5 kPa<br>5.0 kPa | Representative of stiff clay. Value is assumed to increase linearly with burial depth. |
| Angle of shearing resistance of sediment | 20° | Representative of stiff clay |
| Specific weight of sediment | 20 $kN/m^3$ | Representative of stiff clay |
| Dilation angle of sediment | 0° | Representative of stiff clay |
| Young's modulus of carapace and ridge | 2000 MPa | Wet chitin (Hepburn, 1972) |
| Carapace thickness | 0.1 mm | Conservative (low) estimate |
| Carapace dorsal margin thickness | 1 mm | Hinge is assumed to be thicker than the carapace |
| Carapace ventral margin thickness | 1 mm | Small zone near margin modeled as thicker than carapace, to emulate |

| | | |
|---|---|---|
| | | increased stiffness due to out-of-plane curvature at this margin |
| Ridge thickness | 1 mm | Conservative (low) estimate |

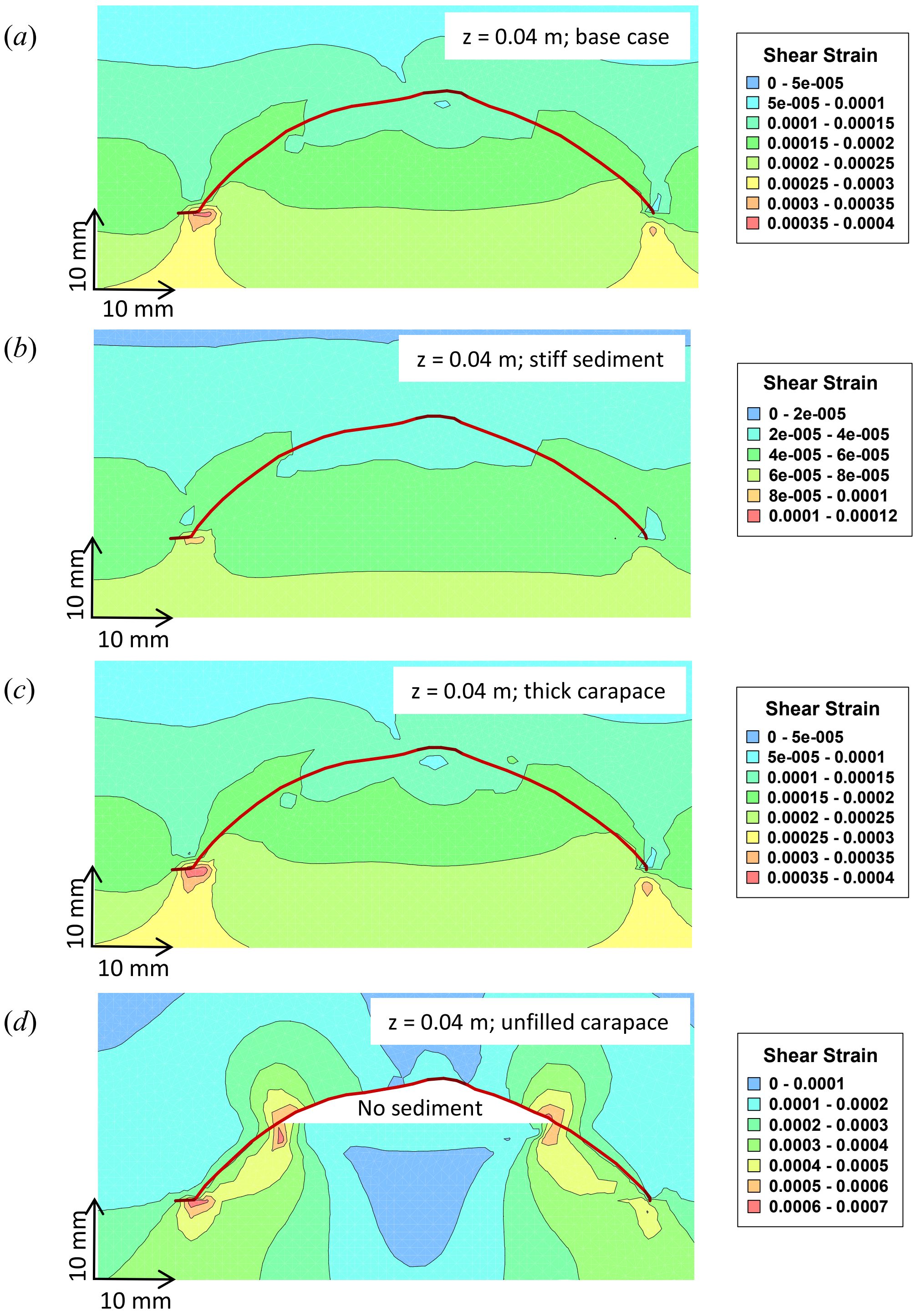

**Figure 1**. Cross-sectional view of *Tuzoia* carapace model geometry. Comparison of shear strain distributions for different carapace scenarios, at 0.04 m burial depth. (*a*) Base case scenario (reformatted version of results shown in figure *8a*). Young's modulus of sediment = 2,700 kPa; carapace thickness = 0.1 mm. Shear strain distribution is approximately symmetrical, with greatest values occurring beneath both margins of the carapace, and lowest values occurring below lateral ridge (peak of carapace) and above carapace margins. (*b*) Stiff sediment scenario (Young's modulus = 10,000 kPa). Distribution of strain is similar to the base case, except strain magnitudes are smaller. (*c*) Thick carapace scenario (thickness = 0.3 mm). Distribution and magnitudes of strain are nearly identical to the base case, except the zones of greatest (and lowest) strain are slightly larger. (*d*) Partially infilled carapace scenario. The general distribution is similar to the base case, though the zones of greatest strain near each margin have grown upwards and inwards (towards to edge of the unfilled cavity), and large zone of low strain exist both above and below the lateral ridge (central peak) of the carapace.
